# The colonic pathogen *Entamoeba histolytica* activates caspase-4/1 that cleaves the pore-forming protein gasdermin D to regulate IL-1β secretion

**DOI:** 10.1371/journal.ppat.1010415

**Published:** 2022-03-18

**Authors:** Shanshan Wang, France Moreau, Kris Chadee

**Affiliations:** Department of Microbiology, Immunology and Infectious Diseases, University of Calgary, Calgary, Alberta, Canada; University of Virginia, UNITED STATES

## Abstract

A hallmark of *Entamoeba histolytica* (*Eh*) invasion in the gut is acute inflammation dominated by the secretion of pro-inflammatory cytokines TNF-α and IL-1β. This is initiated when *Eh* in contact with macrophages in the lamina propria activates caspase-1 by recruiting the NLRP3 inflammasome complex in a Gal-lectin and *Eh*CP-A5-dependent manner resulting in the maturation and secretion of IL-1β and IL-18. Here, we interrogated the requirements and mechanisms for *Eh*-induced caspase-4/1 activation in the cleavage of gasdermin D (GSDMD) to regulate bioactive IL-1β release in the absence of cell death in human macrophages. Unlike caspase-1, caspase-4 activation occurred as early as 10 min that was dependent on *Eh* Gal-lectin and *Eh*CP-A5 binding to macrophages. By utilizing CRISPR-Cas9 gene edited *CASP4/1*, *NLRP3 KO* and ASC-def cells, caspase-4 activation was found to be independent of the canonical NLRP3 inflammasomes. In CRISPR-Cas9 gene edited *CASP1* macrophages, caspase-4 activation was significantly up regulated that enhanced the enzymatic cleavage of GSDMD at the same cleavage site as caspase-1 to induce GSDMD pore formation and sustained bioactive IL-1β secretion. *Eh*-induced IL-1β secretion was independent of pyroptosis as revealed by pharmacological blockade of GSDMD pore formation and in CRISPR-Cas9 gene edited *GSDMD KO* macrophages. This was in marked contrast to the potent positive control, lipopolysaccharide + Nigericin that induced high expression of predominantly caspase-1 that efficiently cleaved GSDMD with high IL-1β secretion/release associated with massive cell pyroptosis. These results reveal that *Eh* triggered “hyperactivated macrophages” allowed caspase-4 dependent cleavage of GSDMD and IL-1β secretion to occur in the absence of pyroptosis that may play an important role in disease pathogenesis.

## Author summary

A unique feature of *Entamoeba histolytica* (*Eh*) infection is the capability to cause symptoms in only a limited subset of individuals. This occurs when *Eh* breaches intestinal innate host defences and comes in contact with the colonic epithelium and immune cells in the lamina propria to elicit a pro-inflammatory response critical in disease pathogenesis. Macrophages are considered among the first responders that *Eh* comes in direct contact with to activate caspase-1 by initiating the assembly of the NLRP3 inflammasome complex in a Gal-lectin and *Eh*CP-A5-dependent manner, resulting in processing and release of IL-1β. In this study, we showed that inflammatory caspase-4 was activated earlier than caspase-1 when *Eh* contacts macrophages independent of the NLRP3 inflammasome complex. More importantly, *Eh*-induced caspase-4 was essential in regulating bioactive IL-1β secretion in the absence of cell death (pyroptosis) that was induced primarily by the activation of caspase-1. Mechanistically, we reveal that *Eh*-induced caspase-4 activation was critically important in regulating a measured amount of gasdermin D (GSDMD) cleavage resulting in GSDMD pore formation that facilitated sustained IL-1β secretion from macrophages. This was in marked contrast to LPS + Nigericin stimulated macrophages that robustly activated casapase-1 via the NLRP3 inflammasome that resulted in almost complete cleavage of GSDMD with pore-forming proteins that caused massive pyroptosis. Our study provides new insights on how *Eh* in contact with macrophages fine tune macrophage responses via the activation of caspase-4/1 to allow the cell to regulate IL-1β release by keeping the cells alive. We believe this mechanism of activating macrophages (termed hyperactivation) is a critically overlooked response in the biology of *Eh* that may play a major role in disease pathogenesis and host defence.

## Introduction

*Entamoeba histolytica* (*Eh*) is a protozoan parasite that infects about 10% of the world’s population resulting in 10^6^ deaths/year [1]. In approximately 90% of infected individuals, *Eh* colonizes the colon and results in a non-invasive and asymptomatic infection [2]. The protective host factors as well as those that contribute to the onset of pathology remain poorly understood. However, under conditions that are not well characterized, *Eh* breaches innate mucosal barriers, disrupts the epithelium and invades the lamina propria and submucosa where it can disseminate through the portal circulation and cause extra intestinal infections. As *Eh* is large, between 20–60 μm in diameter, it is too big to be phagocytosed by neutrophils or macrophages and remains extracellular throughout infection [3].

Macrophages are considered to be essential in the innate immune response to invasive *Eh* by killing the parasite directly and driving a pro-inflammatory response by recruiting inflammatory/immune cells to combat the infection [3,4]. Direct contact of *Eh* with macrophages is a critical “cue” that host cells use to detect *Eh* and initiate host defense [5]. We have shown that the major *Eh* surface adhesin, the Gal/GalNAc lectin (Gal-lectin) mediates an “adhesive” signal at the intercellular junction with macrophages to activate the NLRP3 inflammasome [5,6]. In addition to surface Gal-lectin, the *Eh* genome encodes numerous genes for cysteine proteases (CPs) that play important roles in *Eh* virulence and invasiveness [7,8]. When *Eh* contacts macrophage, it activates caspase-1 by the recruitment of the NLRP3 inflammasome complex in a Gal-lectin and *Eh* cysteine proteases 5 (*Eh*CP-A5)-dependent manner, resulting in the maturation and secretion of interleukin (IL)-1β and IL-18 [5,6]. Inflammasomes are a group of multi-protein cytosolic receptors that are formed to mediate host immune responses to microbial infection and cellular damage [9]. Upon activation by pathogen or damage associated molecular patterns (PAMPs and DAMPs), the nucleotide-binding oligomerization domain (NOD)-like receptor-pyrin containing 3 (NLRP3) signaling triggers oligomerization of the adaptor protein apoptosis-associated speck-like protein (ASC) and the effector pro-caspase-1, resulting in the formation of the inflammasome complex to activate caspase-1 [10]. Activated caspase-1 initiates pro-inflammatory responses by processing the intracellular pro-forms of IL-1β and IL-18 to mediate their maturation and release, along with several other pro-inflammatory mediators, by an undefined secretion step and ultimately causing cell pyroptosis [10–12].

Pyroptosis (fiery death) is a potent inflammatory mode of lytic programmed cell death triggered by cytosolic sensing of diverse infectious and sterile insults that mediates the cleavage of gasdermin D (GSDMD) [13–15]. GSDMD consists of a highly conserved N-terminal (NT) and a C-terminal (CT) components that are connected by a variable linker [16–19]. In the resting state, this pore-forming activity conducted by the NT is held in check by the CT [20], termed the repressor domain (RD) [21]. Caspase-1, caspase-4 (human), caspase-11 (mouse), and caspase-5 (human) are the major effectors of GSDMD cleavage. Upon proteolytic cleavage within the linker region, the CT separates from the NT, and the liberated NT pore-forming fragment of GSDMD oligomerizes into a ring-shaped structure and interacts with acidic phospholipids in the inner leaflet of cell membranes [16–19]. Approximately 16 monomers oligomerize to form a gasdermin pore that mediates pro-inflammatory cytokine release and to elicit cell pyroptosis [17,19,20,22], characterized by the loss of cell membrane integrity, release of cytoplasmic contents and organelles and formation of large ballooning bubbles that are larger than apoptotic blebs [23,24]. Recent studies have advanced a non-pyroptotic role for GSDMD pore-forming activity where phagocytes retain their functions while performing pore-forming activity without causing membrane rupture and pyroptotic cell death, termed “hyperactivated” cells [25]. At present, it is unclear what determines whether cleaved GSDMD plays a pyroptotic or a non-pyroptotic role. Perhaps viability of the cell may reflect how efficiently and rapidly the damaged membrane is repaired depending on the intensity of the damage [26].

Until recently, most work on the canonical NLRP3 inflammasome has been mainly focused on the functions of caspase-1 [27–31]. The non-canonical caspase-4/5/11 inflammasome is considered inevitably connected to the canonical NLRP3 inflammasome. However, the exact mechanisms regulating the interaction between the canonical NLRP3 and non-canonical caspase-4/5/11 inflammasomes and how the activation of the noncanonical inflammasome is regulated remain poorly understood. Caspase-4 is crucial in inflammation by regulating IL-1α, IL-18, IL-8, and MIP-1 secretions and cell death [32–34]. We have recently uncovered that similar to caspase-1, activation of caspase-4 involved potassium (K^+^) efflux and the generation of reactive oxygen species (ROS) [35], and that caspase-4 interacted with caspase-1 in a protein complex to enhance the cleavage of caspase-1 CARD domains [35]. Other studies have revealed that caspase-11, the murine ortholog of caspase-4, is also involved in a non-canonical inflammasome pathway to activate caspase-1 [36,37], suggesting that caspase-4 probably have comparable roles. *Eh*-induced host cell caspases play an important role in disease pathogenesis. *Eh* in contact with Jurkat T cells induces apoptosis [38] and this may occur when *Eh* contact other cell types in the pathogenesis of intestinal amebiasis. In contrast, intracellular parasites inhibit apoptosis to promote host cell survival by targeting caspase activation [39,40].

At present, it is unclear how caspase-4 is activated in response to *Eh* and whether caspase-4/1 act independently or in concert to regulate the cleavage of GSDMD in the absence of significant cell death. In this study, we interrogated the requirements and mechanisms of *Eh*-induced caspase-4 activation that cleaves GSDMD to induce pore-forming proteins that regulates IL-1β secretion in the absence of inflammatory cell death (pyroptosis). Here we highlight an essential role for *Eh*-induced caspase-4 activation that regulates the cleavage of GSDMD in the absence of caspase-1 to mediate sustained IL-1β release from “hyperactivated macrophages” critical in *Eh* disease pathogenesis.

## Results

### *E*. *histolytica*-macrophage interaction regulates the activation of caspase-4

**Fig 1:**
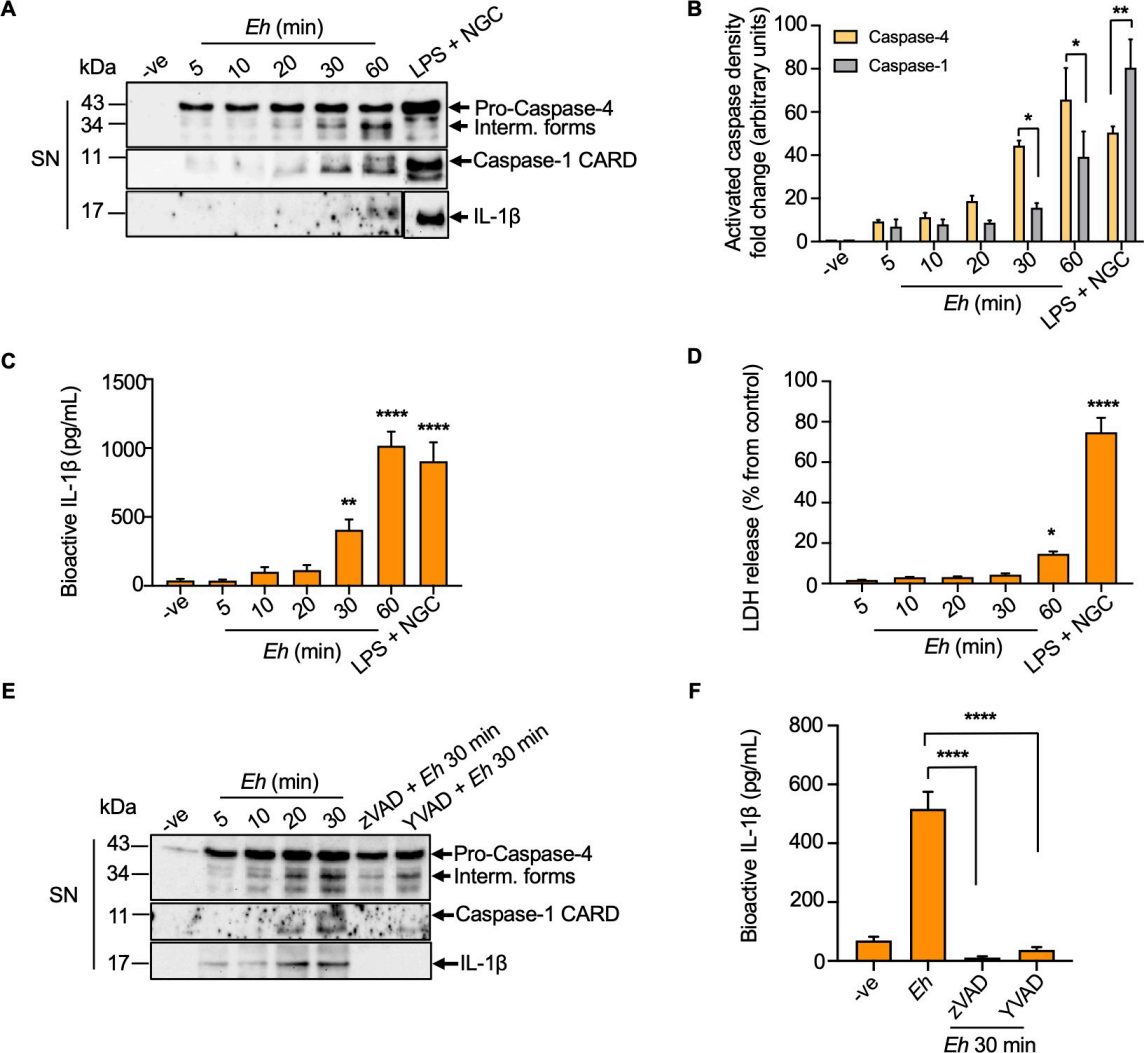
*E*. *histolytica* activates caspase-4 and caspase-1 in a time-dependent manner. The kinetics of *Eh*-induced caspase-4 activation was detected by incubating PMA-differentiated THP-1 macrophages for increasing amounts of time with 1:20 *Eh* to macrophage ratio. (**A**) Macrophages were incubated for increasing amounts of time with 1:20 *Eh* to macrophage ratio. Lipopolysaccharide (LPS) (50 ng/mL) and nigericin (NGC) (10 μM) stimulation for 60 min was used as a positive control. Immunoblot analysis was performed for caspase-4 and caspase-1 in supernatants (SN). (**B**) Quantifications of activated caspase-4 and caspase-1 were performed by densitometric analysis from three independent experiments and the negative control (cells only) acted as an internal control. Statistical significance was calculated between caspase-4 and caspase-1 at each time point. (**C**) Cell free supernatant was added to HEK-Blue IL-1β reporter cells to detect bioactive IL-1β via measurement of SEAP levels. (**D**) Cell death was quantified by lactate dehydrogenase (LDH) release into the culture supernatant and is shown as a percentage of LDH release compared to non-stimulated cells (control). (**E, F**) Macrophages were pre-incubated with the pan-caspase inhibitor Z-VAD-fmk (100 μM) and caspase-1 specific inhibitor Z-YVAD-fmk (100 μM) for 45 min followed by stimulation with *Eh* for 30 min. Caspase-4/1 activation as well as IL-1β secretion in the cell supernatant were assessed via immunoblotting. Cell free supernatant was added to HEK-Blue IL-1β reporter cells to detect bioactive IL-1β using the SEAP assay. Data and immunoblots are representative of at least three experiments (n = 3) and statistical significance was calculated with ANOVA and Bonferroni’s *post-hoc* test (*p < 0.05, **p < 0.01, ****p < 0.0001). Bars represent mean ± SEM.

Direct interaction between *Eh* and macrophage via the Gal-lectin and engagement of *Eh*CP-A5 RGD motif to α_5_β_1_ integrin are important for triggering outside-in signaling to activate the NLRP3 inflammasome characterized by assessing caspase-1 processing and secretion in the extracellular media [5,6]. Activated caspase-1 in turn, cleaves the precursors of IL-1β and IL-18 into bioactive fragments and mediates their release, inducing cell pyroptosis [22]. More recently [35], we discovered that *Eh* activated caspase-4 that interacted with caspase-1 in a protein complex to enhance the cleavage of caspase-1 CARD proteins for IL-1β secretion [5,35]. Unfortunately, we do not know mechanistically how *Eh* activates caspase-4 and to address this deficiency, we first determined the kinetics of caspase-4/1 activation following contact with *Eh* and in response to the positive control lipopolysaccharide (LPS) + Nigericin (NGC) in THP-1 cells. *Eh* activated caspase-4 in a time (**Fig 1A**) and dose-dependent fashion with 1:20 *Eh* to macrophage ratio being the optimal dosage (**S1A Fig**). This time and dosage were used in all subsequent studies. *Eh* activation of caspase-4 was quantified by the appearance of the 30–34 kDa intermediate forms because the cysteine catalytic site was discovered on the large subunit and processing of the pro-form of caspase-4 generates several different intermediate products [41]. Even though both caspase-4/1 were activated a time-dependent fashion in response to *Eh* (**Fig 1A**), unlike caspase-1, higher amount of caspase-4 was activated and released within 10 min of incubation and accumulated in the cell supernatant up until 60 min (**Fig 1B**). These findings suggested that intracellular caspase-4 was cleaved from its inactive pro-form and carried out its bioactive function and subsequently secreted in the cell supernatant prior to caspase-1. Full activation for caspase-1 occurred between 20 and 30 min in response to *Eh* stimulation (**Fig 1A**). In comparison, LPS + NGC vigorously activated and secreted mostly caspase-1 and IL-1β (**Fig 1A and 1B**). Bioactive IL-1β levels gradually increased in a time- and dose-dependent manner, as quantified by HEK-Blue IL-1β reporter cells via the measurement of secreted embryonic alkaline phosphatase (SEAP) (**Figs 1C and S1B**). Unlike LPS + NGC, the release of inflammatory caspases and IL-1β in response to *Eh* (*Eh*-macrophage ratio: 1:20) was not due to significant cellular damage as confirmed by the release of cytosolic lactate dehydrogenase (LDH) into the cell supernatant (**Fig 1D**). Cytosolic LDH release into the extracellular media was used as an indicator for loss of membrane integrity to drive lytic cell death, including pyroptosis, because it is too large to exit through GSDMD NT pores and relies on cell lysis for its secretion. Following prolonged treatment with *Eh* up to 60 min, only 20% of cells death were noted whereas, LPS + NGC killed 80% of the cells as compared to unstimulated controls (**Fig 1D**). As expected, with increasing *Eh* to macrophage ratio, LDH release was significantly increased (**S1C Fig**). To assess whether IL-1β release was caspase-4/1-dependent, macrophages were pretreated with the pan-caspase inhibitor Z-VAD-fmk and the caspase-1-specific inhibitor Z-YVAD-fmk. Inhibition of both caspase-4/1 prevented the maturation and release of IL-1β (**Fig 1E and 1F**). To confirm a role for caspase-4 enzymatic activity in caspase-1 activation, Z-LEVD-FMK was used to inhibit caspase-4 activity in wild type (WT) macrophages stimulated with *Eh* and it also inhibited caspase-1 activation [35]. To test specificity of this inhibitor, CRISPR-Cas9 *CASP4 KO* cells were treated with Z-LEVD-FMK prior to incubation with *Eh* and it also inhibited caspase-1 activation. Based on these finding we did not use this caspase to inhibit caspase-4 [42]. In summary, these data pinpoint the importance of caspase-4/1 in processing and release of IL-1β with low cell death, suggesting that IL-1β was actively regulated and released from macrophages in response to *Eh*.

### Caspase-4/1 activation parallels each other in response to *E*. *histolytica*

**Fig 2:**
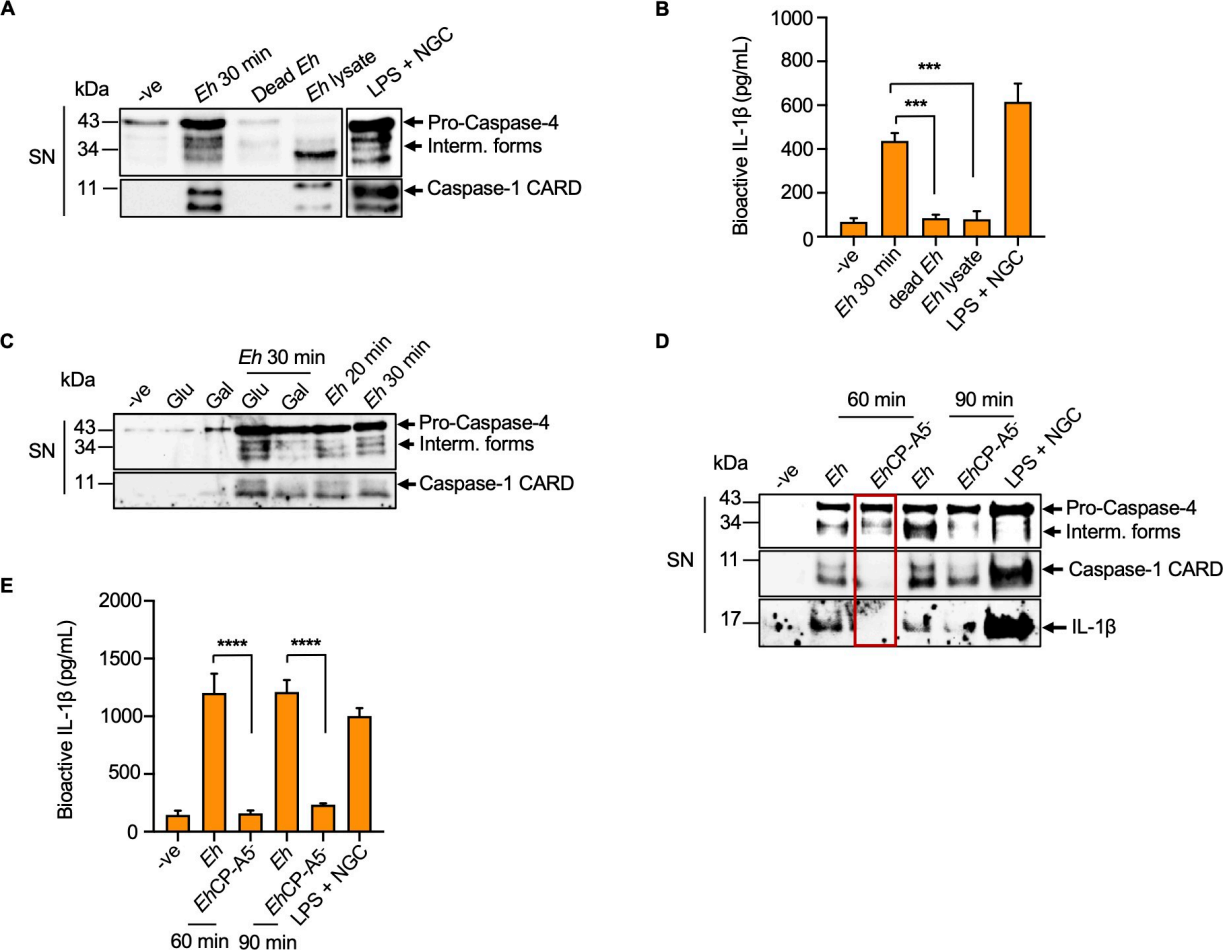
*E*. *histolytica*-macrophage contact is required for caspase-4 activation. (**A, B**) Macrophages were incubated with live *Eh*: macrophage ratio (1:20), fixed *Eh*: macrophage ratio (1:20) for 30 min and with equivalent amount of freeze thawed whole lysates of *Eh*. Live *Eh* were fixed with 1.5% glutaraldehyde for 1 h at 4°C and washed 3 times with sterile cold PBS before use. LPS (50 ng/mL) and NGC (10 μM) stimulation for 60 min acted a positive control. Post incubation, the cell supernatant (SN) was TCA precipitated and equal amount was loaded onto the SDS-PAGE gel to detect caspase-4/1 activation with indicated antibodies. (**B**) Cell supernatant was added to HEK-Blue IL-1β reporter cells to detect bioactive IL-1β using the SEAP assay. Statistical significance was calculated between live *Eh* and fixed (dead) *Eh*, and between live *Eh* and *Eh* lysate (**C**) Macrophages were pretreated for 5 min with 55 mM D-galactose (Gal), or glucose (Glu) as an osmotic control and then incubated with *Eh* for 30 min at a 20:1 ratio. (**D**) Macrophages were incubated with *Eh*, and *Eh* deficient in CP5 (*Eh*CP-A5^-^) for 60 min and 90 min, respectively. LPS (50 ng/mL) and NGC (10 μM) stimulation for 60 min were used as a positive control. (**E**) Cell supernatant was added to HEK-Blue IL-1β reporter cells to detect bioactive IL-1β using the SEAP assay. Statistical significance was calculated between WT *Eh* and *Eh*CP-A5^-^ *Eh*. Data and immunoblots are representative of at least three independent experiments (n = 3) and statistical significance was calculated with one-way ANOVA, followed by Bonferroni’s *post-hoc* test (***p < 0.001, ****p < 0.0001). Bars represent mean ± SEM.

To determine if live *Eh* activated caspase-4 similar to caspase-1, macrophages were stimulated with live *Eh*, dead *Eh*, glutaraldehyde fixed *Eh*, and equivalent amount of freeze thawed whole lysates of *Eh*. As predicted, only live *Eh* in direct contact with macrophages activated caspase-4/1 and induced IL-1β secretion in the cell supernatant (**Fig 2A and 2B**). To define if *Eh* Gal-lectin-mediated adhesion was required for the activation of caspase-4, cells were stimulated with *Eh* in the presence or absence of exogenous galactose that competitively blocked *Eh* from binding to macrophages via the Gal-lectin [43]. Inhibition of contact between *Eh* and macrophages abrogated caspase-4/1 activation as compared to glucose, the osmotic control and *Eh* treatment alone (**Fig 2C**). As caspase-1 activation required both Gal-lectin and *Eh*CP-A5 [5,6], we determined if *Eh*CP-A5^-^ deficient parasites could activation caspase-4 and indeed, it activated less caspase-4 (**Fig 2D**) and IL-1β release as compared to WT *Eh* (**Fig 2E**). These results suggest that *Eh*CP-A5 is required for triggering caspase-4 activation and IL-1β processing.

**Fig 3:**
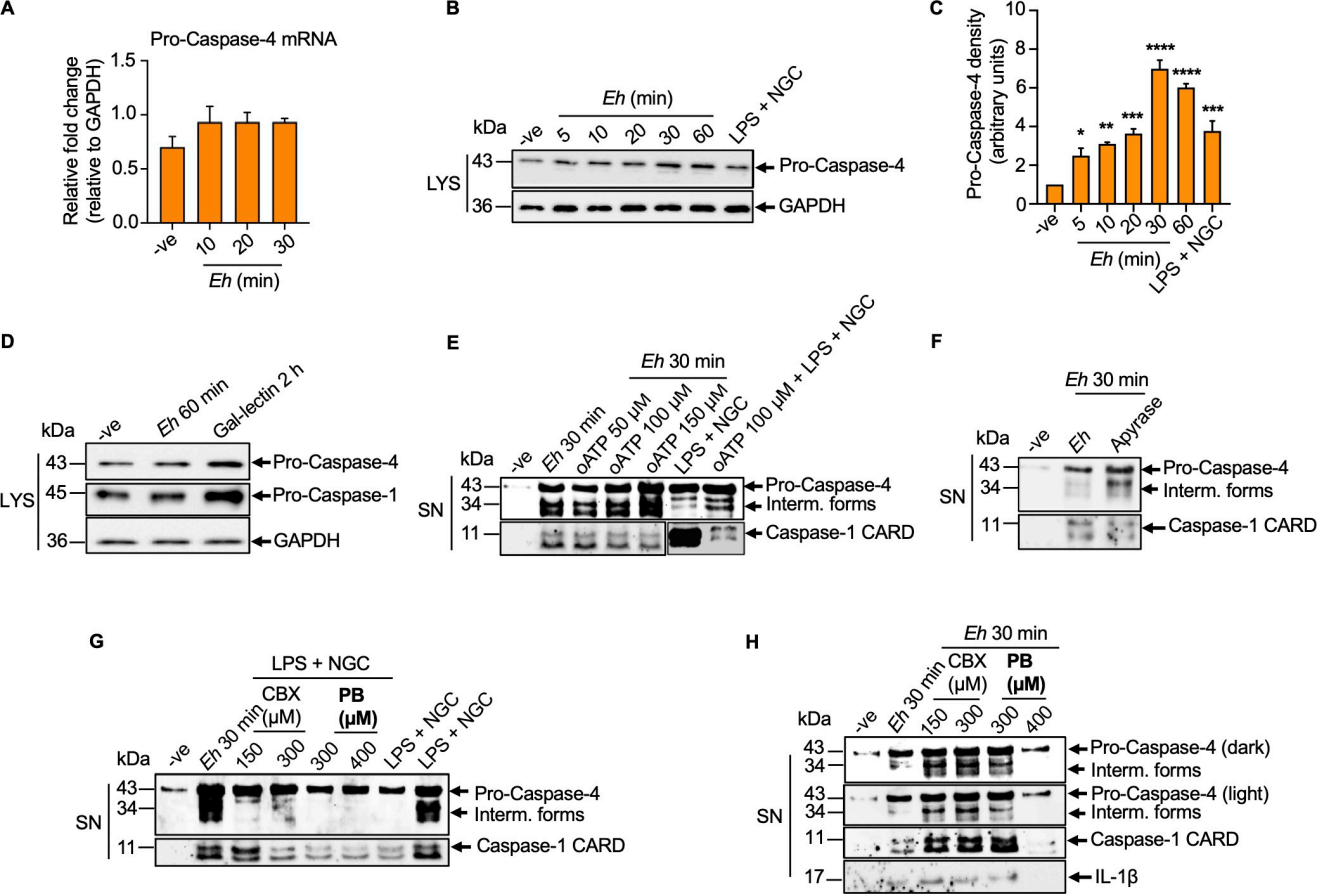
Mechanisms of *E*. *histolytica*-induced caspase-4 activation. (**A-C**) *Eh* upregulated pro-caspase-4 expression and transcription in a time-dependent manner. (**A**) Quantitative PCR was conducted to investigate if *Eh* increased pro-caspase-4 transcription. (**B, C**) Macrophages were stimulated with *Eh* at 20:1 ratio for increasing amount of time. Equal amount of lysate (LYS) was resolved on SDS-PAGE and immunoblotted for pro-caspase-4 detection. Blots were reprobed for GAPDH. Densitometry was performed to assess pro-caspase-4 proteins and the negative (cells only) acted as an internal control. (**D**) Macrophages were treated with native *Eh* Gal-lectin (500 ng/mL) for 2 h and pro-caspase-4, pro-caspase-1 and GAPDH levels were determined by western blot. (**E-H**) Caspase-4 activation requires ATP signaling via the P2X_7_ receptor and pannexin-1 channels. (**E**) Macrophages were pretreated with oxidized ATP (oATP) for 2 h and then stimulated with *Eh* for 30 min. (**F**) Immunoblot analysis of active caspase-4 and caspase-1 in macrophages stimulated for 30 min with *Eh* with the addition of apyrase (20 U/mL). (**G**) Inhibition of caspase-4 with carbenoxolone (CBX), connexin/pannexin channel dual inhibitor, or pannexin channel inhibitor probenecid (PB). LPS and NGC were used as a positive control. Left: LPS (100 ng/ml) priming for 30 min and NGC (5 μM) stimulation for 30 min; right: LPS (50 ng/ml) priming for 30 min and NGC (10 μM) stimulation for 30 min. (**H**) Macrophages were incubated with CBX and PB for 30 min, prior to *Eh* stimulation for 30 min. Cell supernatant (SN) was TCA precipitated and cells were washed and lysed. Equal amount of supernatants and lysed cell lysates was loaded onto SDS-PAGE and immunoblot analysis was performed for caspase-4, caspase-1 and IL-1β. Data and immunoblots are representative of at least three independent experiments (n = 3) and statistical significance was calculated with one-way ANOVA, followed by Bonferroni’s *post-hoc* test (*p < 0.05, **p < 0.01, ***p < 0.001 ****p < 0.0001). Bars represent mean ± SEM.

The activation of caspase-1 requires two-signals to safeguard against unintentional caspase activation. The priming signal upregulates the transcription of NLRP3 and pro-IL-1β, and the second signal recruits NLRP3, ASC, and pro-caspase-1 into a complex for the cleavage of pro-caspase-1 into active caspase-1 [44–47]. We next interrogated how caspase-4 activation is regulated in response to *Eh* and whether it also requires two-signals for its activation. To determine if a priming signal is necessary for caspase-4 activation, macrophages were exposed to *Eh* for increasing time points and pro-caspase-4 transcription and translation were determined through quantitative PCR (qPCR) and western blot analysis, respectively. Pro-caspase-4 transcription (**Fig 3A**) was upregulated within 10 mins and protein expression significantly increased temporally (**Fig 3B and 3C**). Since *Eh* Gal-lectin provides a critical adhesive signal to mediate *Eh*-macrophage attachment, we determined if soluble native Gal-lectin could initiate the priming step to upregulate caspase-4 expression in macrophages. Native Gal-lectin significantly enhanced pro-caspase-4/1 expression after 2 h exposure as compared to *Eh* stimulation for 60 min (**Fig 3D**). These results support the hypothesis that *Eh*-induced caspase-4 activation required binding of Gal-lectin to macrophages as a priming signal that resulted in the upregulation of pro-caspase-4 transcription and expression. As ATP-P2X_7_ receptor signaling is required for caspase-1 activation in response to *Eh* [5], we next investigated whether ATP gated P2X_7_ receptor signaling was required for the activation of caspase-4. To determine if *Eh*-induced ATP release activated caspase-4 in an autocrine fashion similar to caspase-1 [5], macrophages were pre-treated with the specific antagonists of the P2X_7_ receptor, oxidized ATP (oATP), prior to incubation with *Eh*. Surprisingly, increasing the concentrations of oATP upregulated caspase-4, whereas caspase-1 activation was inhibited (**Fig 3E**). To better define the role of ATP in mediating the activation of caspase-4, macrophages were cultured with apyrase that hydrolyses ATP to AMP and PPi. Upon addition of apyrase, the activation of caspase-4 was increased with a corresponding decrease in caspase-1 activation in response to *Eh*. These data validate the importance of ATP in activating caspase-4, and in cells treated with apyrase, caspase-4 activation was increased indicating that ATP acted as a second signal to induce its activation (**Fig 3F**). Given that the P2X_7_ receptor is involved in mediating the activation of caspase-4 in response to *Eh*, and ATP is conducted into the extracellular space by non-junctional (hemi) pannexin-1 channels, we next delineated if pannexin-1 channels was required for the activation of caspase-4. To test whether *Eh*-induced ATP release through either the pannexin-1 or connexin channels [5,48] activated caspase-4, two inhibitors were used to block the channels, carbenoxolone (CBX), a dual antagonist of connexin/pannexin channels, and the specific pannexin antagonist, probenecid (PB). PB abolished caspase-4/1 activation and inhibited IL-1β release following stimulation with either LPS + NGC (**Fig 3G**) or *Eh* (**Fig 3H**), indicating that the release of ATP through the pannexin-1 channels is a critical signal for activating caspase-4. These results confirm that ATP acted as the second signal through the pannexin-1 channels to signal back onto the P2X_7_ receptor to activate caspase-4 in response to *Eh*.

Ligation of *Eh*CP-A5 RGD sequence to α_5_β_1_ integrin on macrophage is an essential trigger to induce ATP release to activate the NLRP3 inflammasome [5]. To quantify the function of ATP as a potential second signal for *Eh*-induced caspase-4 activation, we added exogenous ATP to both WT and CRISPR-Cas9 *CASP1 KO* cells for the indicated time points. *Eh* stimulation alone was used as a positive control. Exogenous ATP modestly rescued caspase-4 activation in CRISPR-Cas9 *CASP1 KO* but not in WT macrophages; inflammasome activation was not restored (**S2A and S2B Fig**). To address if *Eh*CP-A5 RGD sequence induces ATP release to activate the NLRP3 inflammasome is the singular function of *Eh*CP-A5, we applied exogenous ATP to macrophage culture following stimulation with *Eh*CP-A5^-^ *Eh*. Immunoblot analysis of secreted active caspase-4/1 cleavage product was performed and IL-1β release was quantified by SEAP assay from macrophages stimulated with WT *Eh* or *Eh*CP-A5^-^ *Eh* for 60 or 90 min, with or without the addition of exogenous ATP, respectively (**S2C and S2D Fig**). Strikingly, exogenous ATP modestly restored caspase-4 activation and rescued IL-1β maturation and secretion in CRISPR-Cas9 *CASP1* cells in response to *Eh*CP-A5^-^ *Eh*, whereas it did not restore inflammasome activation (caspase-1 activation and IL-1β release) with *Eh*CP-A5^-^ *Eh* in both cell types (**S2E and S2F Fig**). These data indicate that *Eh*CP-A5^-^ initiates additional signaling that is critical for caspase-4 and NLRP3 inflammasome activation.

### Caspase-4 activation does not require the NLRP3 inflammasome assembly in response to *E*. *histolytica*

**Fig 4:**
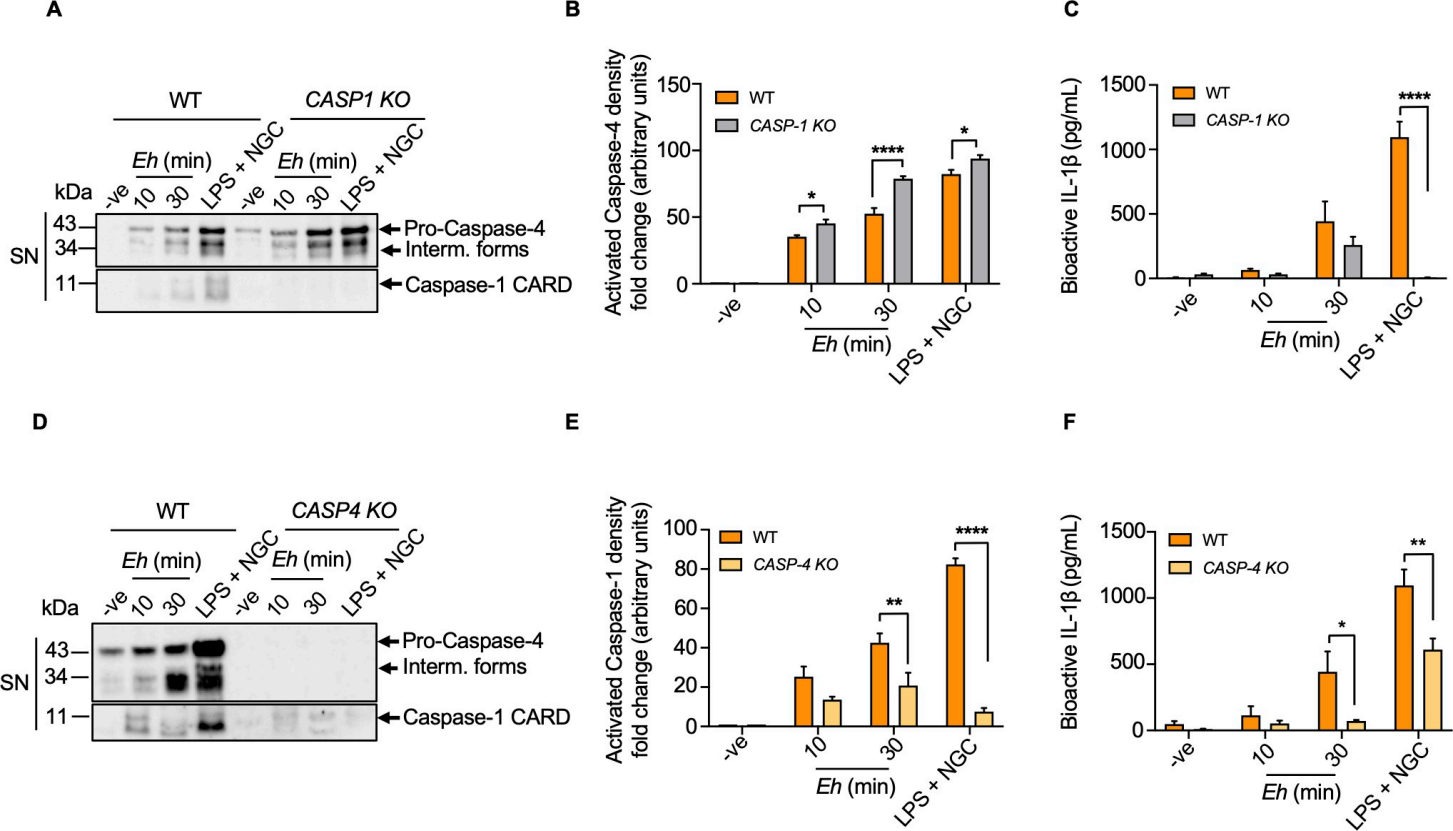
*E*. *histolytica*-induced caspase-4 activation is enhanced in the absence caspase-1. (**A, B**) WT and CRISPR/Cas9 *CASP1 KO* macrophages were stimulated with *Eh* for 10 and 30 min and unstimulated cells were used as an internal control. Macrophages stimulated with LPS (50 ng/mL) and NGC (10 μM) was used as a positive control. Quantifications of active caspase-4 protein were performed by densitometric analysis and negative (cells only) acted as an internal control. Statistical significance was calculated between WT and *CASP1 KO* macrophages at each time point (**C**) Bioactive IL-1β secretion in the histogram was quantified by the SEAP assay and statistical significance was calculated between WT and *CASP1 KO* macrophages at each time point. (**D, E**) WT and CRISPR/Cas9 *CASP4 KO* macrophages were incubated with *Eh* (20:1) at increasing time points. Caspase-1 CARD densitometry was measured and statistical significance was calculated between WT and *CASP4 KO* macrophages at each time point. Cell supernatant (SN) was TCA precipitated and equal amount of cell supernatants was loaded onto SDS-PAGE and immunoblot analysis was performed for caspase-4 and caspase-1. Active caspase-1 was quantified by densitometric analysis, and negative (cells only) acted as an internal control. (**F**) Cell supernatant was added to HEK-Blue IL-1β reporter cells to detect bioactive IL-1β secretion via measuring the SEAP and statistical significance was calculated between WT and *CASP4 KO* macrophages at each time point. Data and immunoblots are representative of at least three separate experiments (n = 3) and statistical significance was calculated with with Student’s t-test between *KO* and WT, (*p < 0.05, **p < 0.01, ****p < 0.0001). Bars represent mean ± SEM.

We next quantified if *Eh*-induced caspase-4 activation is regulated via the canonical NLRP3 inflammasome, since the non-canonical caspase-4 inflammasome is considered inextricably linked to the canonical NLRP3 inflammasome, but crosstalk between these two pathways remains unclear. To dissect if *Eh*-induced caspase-4 activation is regulated by different NLRP3 inflammasome components, we first investigated if caspase-1 was required for *Eh*-induced caspase-4 activation. WT and CRISPR/Cas9 *CASP1 KO* macrophages were incubated with *Eh* for 10 and 30 min using LPS + NGC as a positive control. Unexpectedly, caspase-4 activation but not bioactive IL-1β secretion was significantly enhanced in *CASP1 KO* macrophages, indicating that caspase-4 activation was independent of caspase-1 in response to *Eh* (**Fig 4A–4C**). Caspase-4 activation was also enhanced in response to the positive control, LPS + NGC (**Fig 4B**). To interrogate whether caspase-1 was dependent on caspase-4 for its activation, CRISPR/Cas9 *CASP4 KO* macrophages were stimulated with *Eh* for 10 and 30 min. Intriguingly, caspase-1 activation (**Fig 4D and 4E**) and IL-1β secretion (**Fig 4F**) were significantly decreased in *CASP4 KO* macrophages as compared to WT counterparts, suggesting a critical role of caspase-4 in regulating caspase-1 activation in response to *Eh*. Consistent with *Eh* stimulation, LPS + NGC exhibited vigorously less caspase-1 activation and IL-1β secretion in *CASP4 KO* cells. Collectively, these data revealed that caspase-4/1 interacted to synergize pro-inflammatory responses elicited by *Eh*.

**Fig 5:**
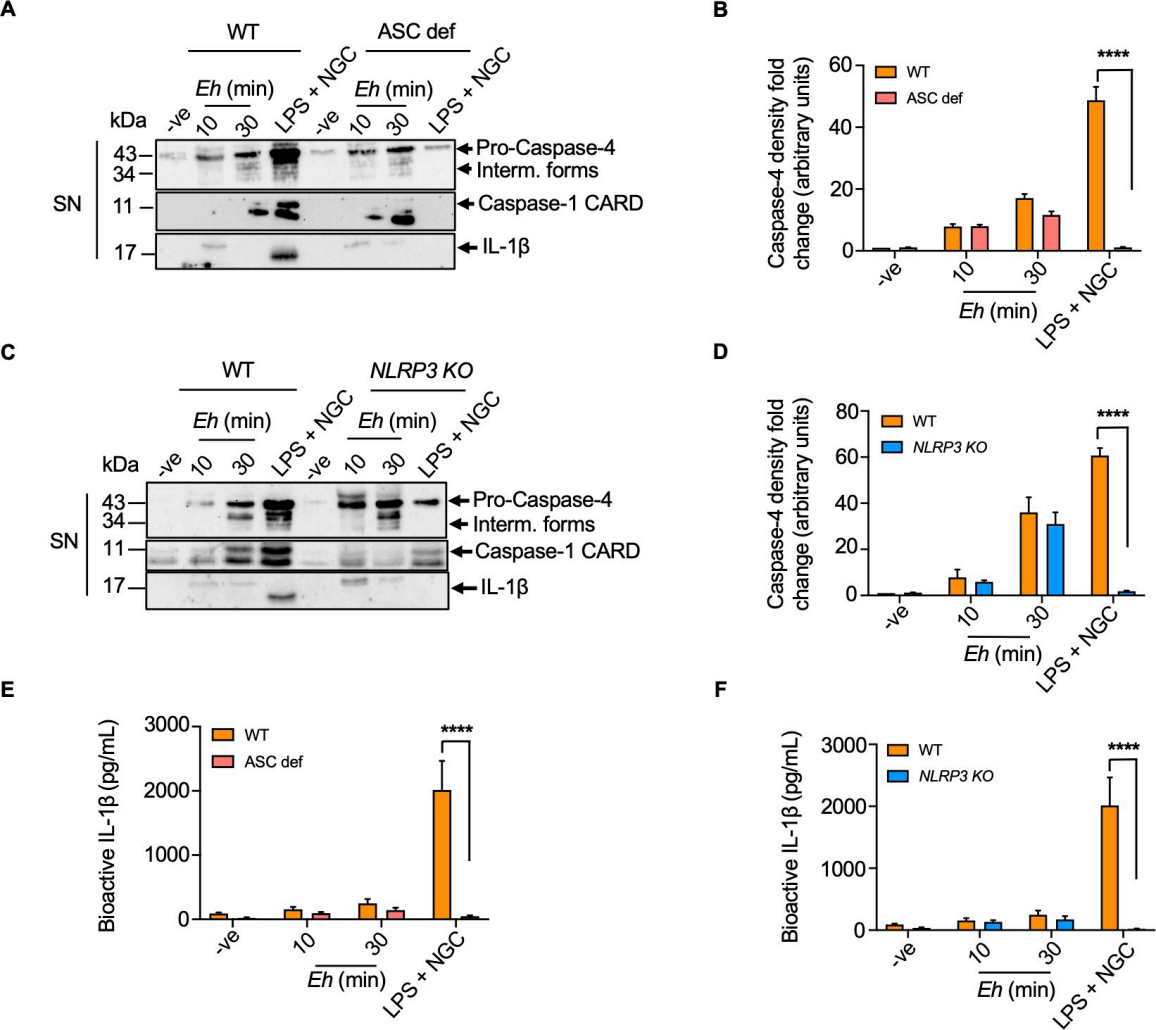
*E*. *histolytica*-induced caspase-4 activation does not require the recruitment of the canonical inflammasome components. (A, B) WT and ASC deficient (ASC def) macrophages were treated with *Eh* (20:1) for 10 min and 30 min, respectively. Macrophages stimulated with LPS (50 ng/mL) and NGC (10 μM) acted as a positive control. *Eh*-induced caspase-4 activation was independent of ASC confirmed by aborted caspase-1 activation and IL-1β secretion. Quantifications of active caspase-4 were performed by densitometric analysis and statistical significance was calculated between WT and ASC def macrophages at each time point. (C, D) WT and CRISPR/Cas9 *NLRP3 KO* macrophages were stimulated with *Eh* (20:1) at 10 min and 30 min, respectively. Macrophages stimulated with LPS (50 ng/mL) and NGC (10 μM) were used as a positive control. Quantifications of activated caspase-4 were confirmed with densitometric analysis and statistical significance was calculated between WT and *NLRP3 KO* macrophages at each time point. Cell supernatant (SN) was TCA precipitated and equal amount of cell supernatants was loaded onto SDS-PAGE and immunoblot analysis was performed for caspase-4, caspase-1 and IL-1β. (E, F) Bioactive IL-1β secretion in the cell supernatant was quantified by the SEAP assay in HEK-Blue reporter cells and statistical significance was calculated between WT and ASC def, WT and *NLRP3 KO* macrophages at each time point. Data and immunoblots are representative of at least three independent experiments (n = 3) and statistical significance was calculated with Student’s t-test between *KO* and WT (****p < 0.0001). Bars represent mean ± SEM.

The mechanisms governing crosstalk between the NLRP3 sensor and caspase-4/5/11 in response to *Eh* remains poorly understood. As the NLRP3 inflammasome requires the recruitment of ASC, NLRP3, and pro-caspase-1 into a high multimeric complex for activating caspase-1, we determined if the NLRP3 inflammasome components are also involved in activating caspase-4. To do this, macrophages deficient in ASC (ASC def) were stimulated with *Eh* and caspase-4 activation was unaffected, whereas, less caspase-1 CARD proteins and IL-1β secretion were evident in ASC def macrophages (**Fig 5A and 5B**). Similarly, in CRISPR/Cas9 *NLRP3 KO* macrophages, caspase-4 activation was not significantly decreased (**Fig 5C**). However, there was less caspase-1 CARD proteins and IL-1β secretion as compared to WT macrophages, supporting the notion that NLRP3 is the central inflammasome activated by *Eh* to regulate IL-1β maturation and release (**Fig 5C and 5D**). These data demonstrate that caspase-4 activation is not dependent on the components of canonical NLRP3 inflammasome in response to *Eh*. *Eh* stimulation of macrophages for 30 mins did not cause significant bioactive IL-1β secretion in ASC def and *NLRP3 KO* macrophages as compared to WT (**Fig 5E and 5F**). This was in marked contrast to LPS + NGC stimulated WT cells were caspase-1 activation and IL-1β release were prominent, supporting the notion that LPS + NGC is highly dependent on the NLRP3 inflammasome pathway to elicit pro-inflammatory responses.

### *E*. *histolytica*-induced caspase-4/1 cleaves gasdermin D to mediate IL-1β release

**Fig 6:**
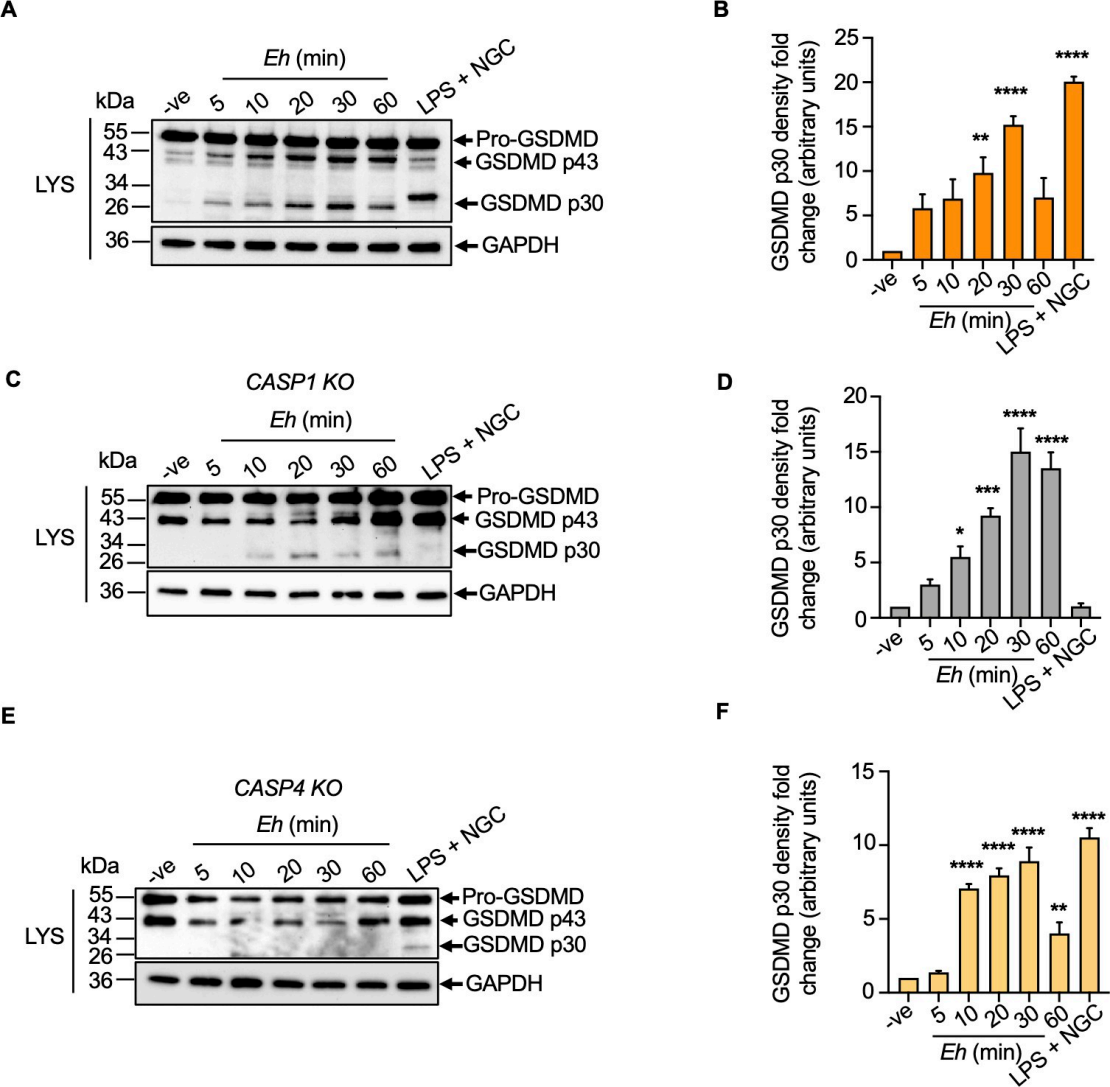
*E*. *histolytica* activates caspase-4 and caspase-1 in a time-dependent manner and cleaves gasdermin D to regulate IL-1β secretion. (**A, C, E**) WT, CRISPR/Cas9 *CASP1 KO*, and CRISPR/Cas9 *CASP4 KO* macrophages were incubated for increasing amounts of time with 1:20 *Eh* to macrophage ratio. LPS (50 ng/mL) and NGC (10 μM) for 60 min acted as a positive control. Cells were washed, lysed and equal amount of lysates (LYS) was loaded onto SDS-PAGE and immunoblot analysis was performed for GSDMD cleavage that present in cell lysates. (**B, D, F**) Quantifications of GSDMD p30 protein were performed by densitometric analysis and blots were reprobed for GAPDH. Negative cells only acted as an internal control. Data and immunoblots are representative of at least three independent experiments (n = 3) and statistical significance was calculated using with an ANOVA and Bonferroni’s *post-hoc* test (*p < 0.05, **p < 0.01, ***p < 0.001, ****p < 0.0001). Bars represent mean ± SEM.

Proteomics have identified GSDMD as the most efficient and selective substrate for inflammatory caspases [49]. The function of GSDMD and its cleavage remained unclear until the investigation of GSDMD as the key executor of inflammasome-induced pyroptosis was uncovered using a chemical mutagenesis screen in mice and a cell-based CRISPR screen [20,50]. In these studies, GSDMD was identified as the critical mediator for pyroptotic cell death initiated by caspase-1/11 activation [20,22,50]. However, caspase-4 as the human ortholog of caspase-11 was less characterized, especially in the context of parasitic infections. To explore this, we first investigated the kinetics of *Eh*-induced caspase-4/1 activation in cleaving GSDMD and IL-1β release. Macrophages were incubated with *Eh* for increasing amount of time and immunoblot analysis demonstrated that as early as 5 min, the 55 kDa GSDMD pro-form was cleaved into the NT p30 pore-forming fragment that accumulated steadily in a time-dependent manner that peaked at 30 min (**Fig 6A and 6B**). This time frame perfectly paralleled the activation of caspase-4/1 (**Fig 1A and 1B**). As *Eh*CP-A5 is required for caspase-4/1 activation and IL-1β processing, we next explored whether GSDMD cleavage would be affected by stimulating macrophages with *Eh*CP-A5^-^*Eh* as compared WT *Eh*. Surprisingly, *Eh*CP-A5^-^*Eh* significantly enhanced the cleavage of GSDMD but not IL-1β secretion as compared to WT *Eh* (**S3A–S3C Fig**). This unexpected finding hinted that other caspases or cysteine proteases might be involved in cleaving GSDMD. In support of this, we have found that both *Eh*CP-A1 and *Eh*CP-A4 were polarized to the site of contact with macrophages to initiate rapid caspase-6-dependent degradation of the cytoskeletal-associated proteins, paxillin, talin, and Pyk2 to induce downstream inflammatory signaling pathways [51].

To determine if activated caspase-4 cleaved GSDMD, CRISPR/Cas9 *CASP1 KO* macrophages were stimulated with *Eh* and GSDMD was cleaved into the p30 NT fragment in a time-dependent manner, whereas, in LPS + NGC treated cells there was no cleavage of GSDMD suggesting complete inhibition of the NLRP3 inflammasome (**Fig 6C and 6D**). In *Eh* stimulated CRISPR/Cas9 *CASP4 KO* cells, GSDMD cleavage was markedly inhibited but was maintained in the positive control, LPS + NGC (**Fig 6E and 6F**). Similarly, cleavage of GSDMD was inhibited with the pannexin channel inhibitor, probenecid (PB) that blocked caspase-4 activation (**Figs S4A, S4B,** **3G and 3H**). The pan-caspase inhibitor, Z-VAD-fmk and caspase-1 inhibitor, Z-YVAD-fmk also inhibited the cleavage of GSDMD cleavage by inhibiting both caspase-4/1 (**S4C and S4D Fig**). These results support a crucial role for activated caspase-4 in cleaving GSDMD in response to *Eh*. These findings are noteworthy as previously it was widely considered that only caspase-1/11 triggered the cleavage of GSDMD and this study shows clearly that caspase-4 can also promote the cleavage of GSDMD. Even though both canonical and non-canonical inflammasomes were discovered to mediate the cleavage of GSDMD [50], it is unclear how inflammatory caspases regulate GSDMD cleavage upon *Eh* stimulation, and if the cleaved products can even impact the activity of upstream effectors.

### Caspase-4 is essential for gasdermin D-regulated pro-inflammatory cytokine release independent of the NLRP3 inflammasome in response to *E*. *histolytica*

**Fig 7:**
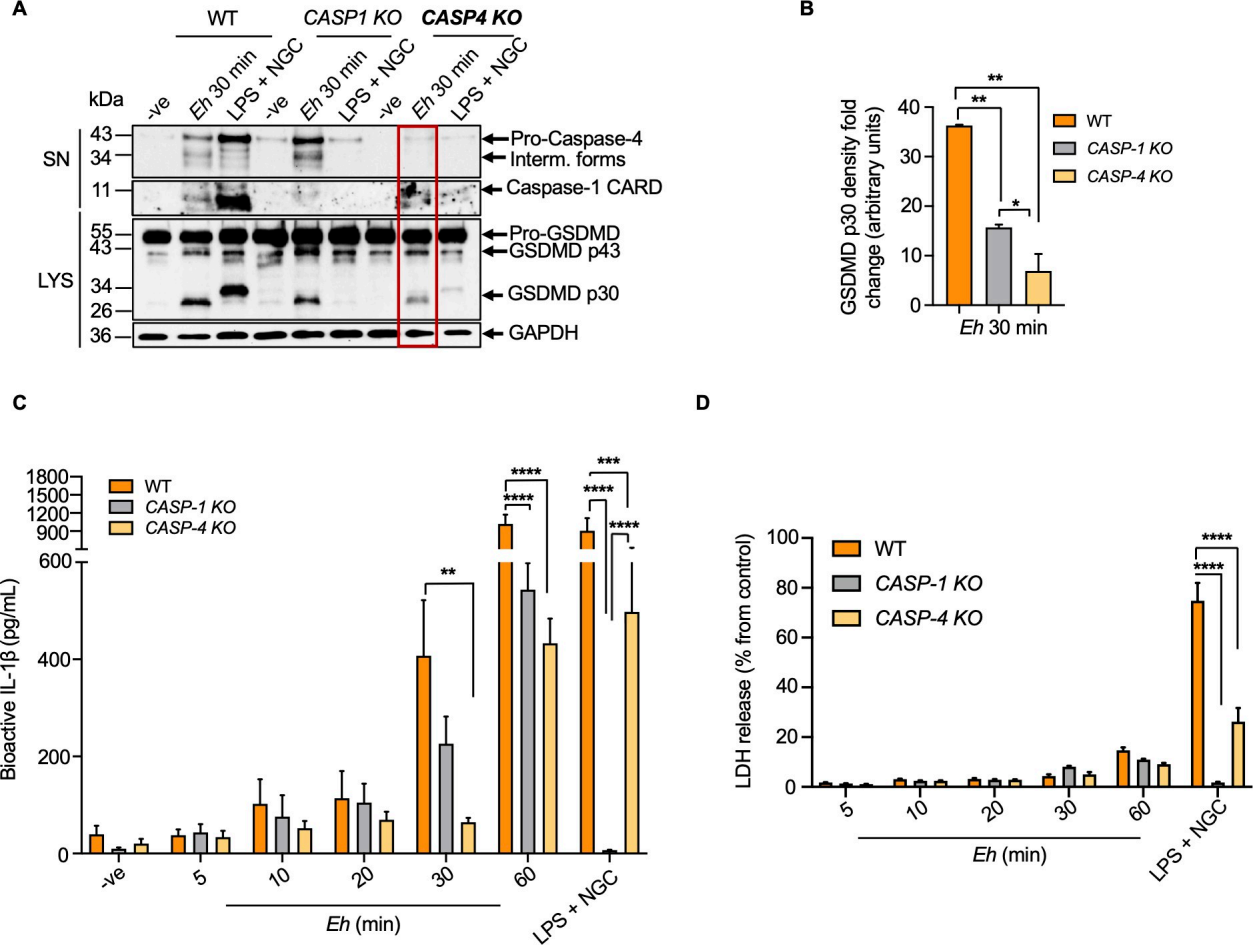
Dependency for *E*. *histolytica*-induced caspase-4 in gasdermin D cleavage and bioactive IL-1β release. (**A**) WT, CRISPR/Cas9 *CASP1 KO* and CRISPR/Cas9 *CASP4 KO* macrophages were incubated with *Eh* for 30 min or the positive control LPS (50 ng/mL) and NGC (10 μM) for 60 min. Cell supernatant (SN) was TCA precipitated and cells were washed and lysed. Equal amount of supernatants and lysates (LYS) was loaded onto SDS-PAGE and immunoblot analysis was performed for caspase-4 and caspase-1 secreted to the supernatants and along with GSDMD p30 pore-forming fragment in lysates, and blots were reprobed for GAPDH. (**B**) Quantifications of cleaved GSDMD were performed by densitometric analysis from three independent experiments, and the negative (cells only) acted as an internal control. (**C**) Cell supernatant was added to HEK-Blue reporter cells to detect bioactive IL-1β by measuring SEAP. (**D**) THP-1 cell supernatant was assessed by the release of LDH following *Eh* stimulation and normalized to non-stimulated negative controls (basal cell death). Data and immunoblots are representative of at least three independent experiments (n = 3) and statistical significance was calculated with ANOVA and Bonferroni’s *post-hoc* test between WT and *KO* macrophages and between *CASP1KO* and *CASP4 KO* macrophages at each time point. (*p < 0.05, **p < 0.01, ***p < 0.001, ****p < 0.0001). Bars represent mean ± SEM.

To determine which caspase was more important in cleaving GSDMD, CRISPR/Cas9 *CASP1 KO* and *CASP4 KO* macrophages were stimulated with *Eh* for 30 min using LPS + NGC as a positive control. In the absence of caspase-4/1, cleavage of GSDMD was significantly reduced and the effect was significantly more pronounced in *CASP4 KO* in response to *Eh* (**Fig 7A and 7B**). These results indicate that cleavage of GSDMD was not solely dependent on only one caspase but rather, both caspase-4/1 interacted together for maximal cleavage. Thus, to better define the role of caspase-4/1 in mediating IL-1β release, WT and CRISPR/Cas9 *CASP4/1 KO* were stimulate with *Eh* using LPS + NGC as a potent agonist for IL-1β (**Fig 7C**). Consistent with decreased cleavage of GSDMD in *CASP4 KO* cells, IL-1β release was significantly decreased as compared to WT stimulated with *Eh*. Conversely, there was only marginal reduction in IL-1β secretion in *CASP1 KO* in response to *Eh* and complete inhibition with LPS + NGC. As 30 min was too short to accurately quantify and discern differences in bioactive IL-1β secretion in WT and *CASP4/1 KO* cells, the kinetics of IL-1β release was measured up to 60 mins (**Fig 7C**). As predicted, *Eh*-induced IL-1β secretion occurred in a time-dependent manner with peak secretion after 60 min with significant reduction in IL-1β release in *CASP4/1 KO* cells in the absence of cell death (**Fig 7D**). These data reveal that activation of caspase-4 was essential for cleaving GSDMD to mediate bioactive IL-1β secretion. Intriguingly, *Eh*-induced cleavage of GSDMD was not dependent on caspase-1 activation but rather, may synergize with caspase-4 for enhanced cleavage. The dependency for caspase-4 in regulating IL-1β secretion in response to *Eh* was measured and sustained in the absence of cell death in comparison to the positive control, LPS + NGC that caused excessive IL-1β release and massive pyroptosis (**Fig 7D**).

**Fig 8:**
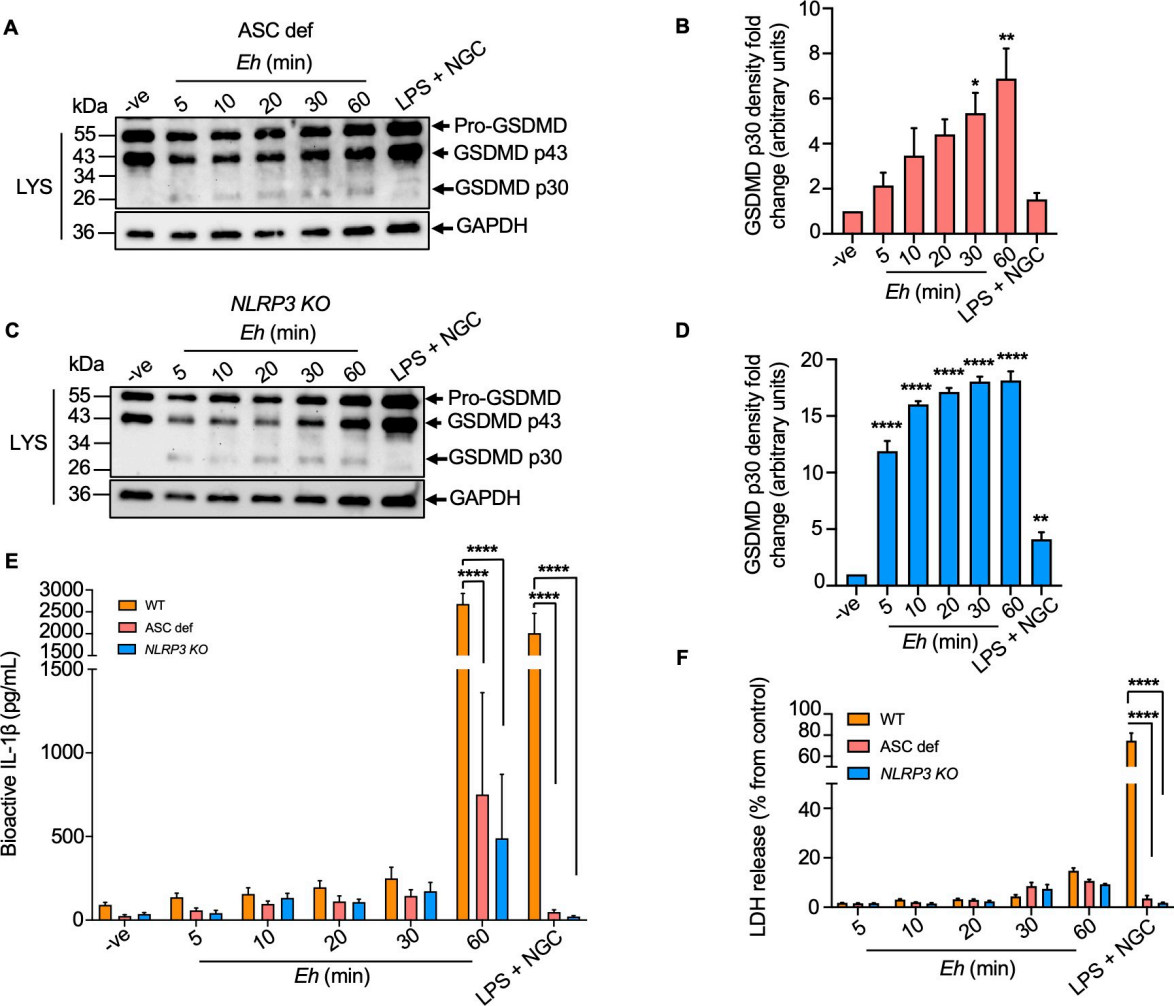
*E*. *histolytica*-induced gasdermin D cleavage is not dependent on the canonical NLRP3 inflammasome components. (**A, C**) ASC def and CRISPR/Cas9 *NLRP3 KO* macrophages were incubated with *Eh* from 5 min to 60 min or the positive control, LPS (50 ng/mL) and NGC (10 μM) for 60 min. Equal amount of cell lysates (LYS) was loaded onto SDS-PAGE and immunoblot analysis was performed for GSDMD p30 pore-forming fragment presented in lysates and blots were reprobed with GAPDH. (**B, D**) Quantification of cleaved GSDMD p30 fragment was performed by densitometric analysis from three independent experiments, and the negative (cells only) acted as an internal control. (**E**) Bioactive IL-1β levels were quantified by SEAP assay that detected in HEK-Blue reporter cells. (**F**) Cell death was quantified by LDH release into the culture supernatant and is shown as a percentage of LDH release compared to non-stimulated cells (control). (**E, F**) Statistical significance was calculated between WT and ASC def, WT and *NLRP3 KO* as well as between ASC def and *NLRP3 KO* macrophages at each time point. Data and immunoblots are representative of at least three independent experiments (n = 3) and statistical significance was calculated with ANOVA and Bonferroni’s *post-hoc* test (*p < 0.05, **p < 0.01, ****p < 0.0001). Bars represent mean ± SEM.

We next substantiated if NLRP3 inflammasome components were indispensably involved in the cleavage of GSDMD. Mechanistically, genetic deficiencies in inflammasome components (e.g., NLRP3 or ASC) prevent caspase-1 activation, resulting in insufficient cleavage of GSDMD and defects in pyroptosis [20]. To determine if *Eh*-induced cleavage of GSDMD requires the recruitment of NLRP3 inflammasome components, ASC def and CRISPR/Cas9 *NLRP3 KO* macrophages were exposed to *Eh* from 5 min to 60 min and the cleaved GSDMD p30 fragment was confirmed via immunoblot analysis. As predicted, comparable GSDMD cleavage was noted in both ASC def and *NLRP3 KO* macrophages (**Fig 8A–8D**) with significant decrease in IL-1β release at 60 min (**Fig 8E**) in the absence of cell death (**Fig 8F**) as compared to WT cells. These results suggest that both the non-canonical caspase-4 and the canonical NLRP3 inflammasome are required to initiate sufficient cleavage of GSDMD to mediate IL-1β secretion, whereas the NLRP3 inflammasome is dispensable in this process.

### Cleavage of gasdermin D regulates IL-1β release in the absence of cell death in response to *E*. *histolytica*

**Fig 9:**
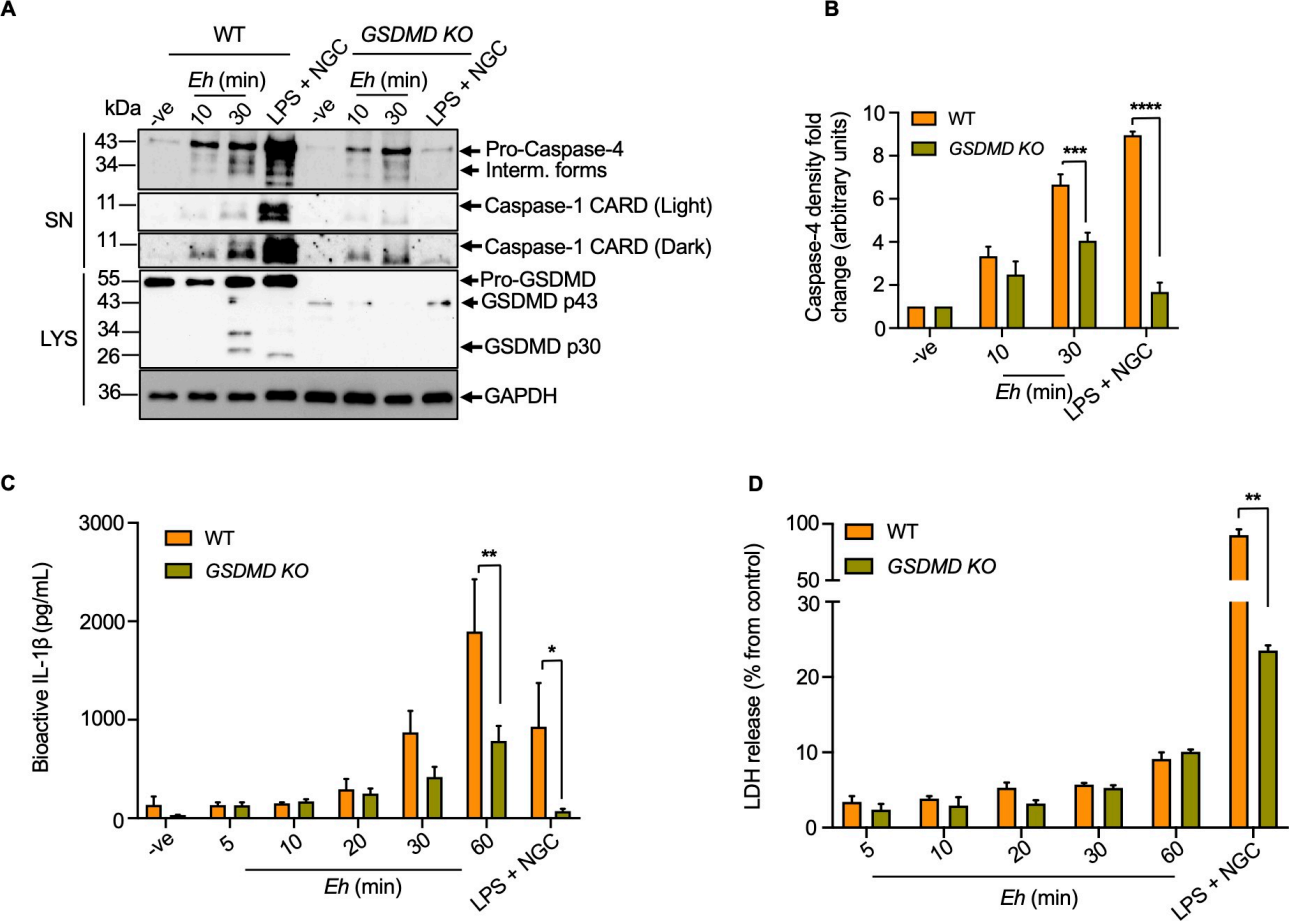
*E*. *histolytica* triggered gasdermin D cleavage regulates bioactive IL-1β release. (**A**) WT and CRISPR/Cas9 *GSDMD KO* macrophages were incubated with *Eh* for 10 and 30 min, respectively. LPS (50 ng/mL) and NGC (10 μM) stimulation for 60 min acted as the positive control. Cell supernatant (SN) was TCA precipitated and cells were washed and lysed. Equal amount of supernatants and lysates (LYS) was resolved on SDS-PAGE and immunoblot analysis was performed for GSDMD, caspase-4 and caspase-1 in both the cell lysates and supernatants, and blots were reprobed for GAPDH. (**B**) Quantifications of active caspase-4 proteins were performed by densitometric analysis from three independent experiments, and the negative (cells only) acted as an internal control. (**C**) Cell supernatant from stimulated macrophages was added to HEK-Blue reporter cells to detect bioactive IL-1β via the SEAP assay in both WT and CRISPR/ Cas9 *GSDMD KO* macrophages that were incubated with *Eh* for increasing amounts of time. (**D**) Cell death was determined by LDH assay using supernatant from stimulated macrophages, and is shown as a percentage of LDH release compared to non-stimulated cells (control). Data and immunoblots are representative of three separate experiments (n = 3) and a one-way ANOVA was used to determine statistical significance of differences between WT and *GSDMD KO* cells at each treatment time (*p < 0.05, **p < 0.01, ***p < 0.001, ****p < 0.0001). Bars represent mean ± SEM.

GSDMD pores breaks the normal permeability barrier of cell membranes to disrupt cellular electrochemical potential to cause cell death. To determine whether GSDMD regulates pore formation and IL-1β release in response to *Eh*, studies were performed using CRISPR/Cas9 *GSDMD KO* macrophages. Intriguingly, even though *GSDMD KO* macrophages preserved the ability to activate the NLRP3 inflammasome in response to *Eh* and LPS + NGC, caspase-4/1 activation (**Fig 9A and 9B**), IL-1β secretion (**Fig 9C**) and pyroptosis (**Fig 9D**) was significantly reduced. As IL-1β release was not completely blocked in *GSDMD KO* cells in response to *Eh* (**Fig 9C**), these results suggest that other secretory pathways could mediate low level of IL-1β release. Cells that release IL-1β in a GSDMD-dependent manner while maintaining viability, reach a state that is different from their activated or pyroptotic counterparts termed “hyperactivated” [25,52]. Our data indicate that *Eh*-stimulated macrophages achieved a “hyperactivated state” with IL-1β release in the absence of excessive cell pyroptosis while maintaining efficient cleavage of GSDMD by active caspase-4/1 (**Fig 9A–9D**). This was in marked contrast to the positive control, LPS + NGC, where caspase-1 was highly activated that enhanced the cleavage of GSDMD to induce high levels of IL-1β and caused massive cell death (**Fig 9C and 9D**). At present, it is unclear if *Eh*-induced activated caspase-4/1 compete with each another to cleave GSDMD or, if the early activation of caspase-4 acted upstream of caspase-1 to regulate its activation and cleave GSDMD or whether, caspase-4 is the major caspase that promote GSDMD pore formation.

### *E*. *histolytica*-induced gasdermin D pores function as a conduit to regulate IL-1β release

**Fig 10:**
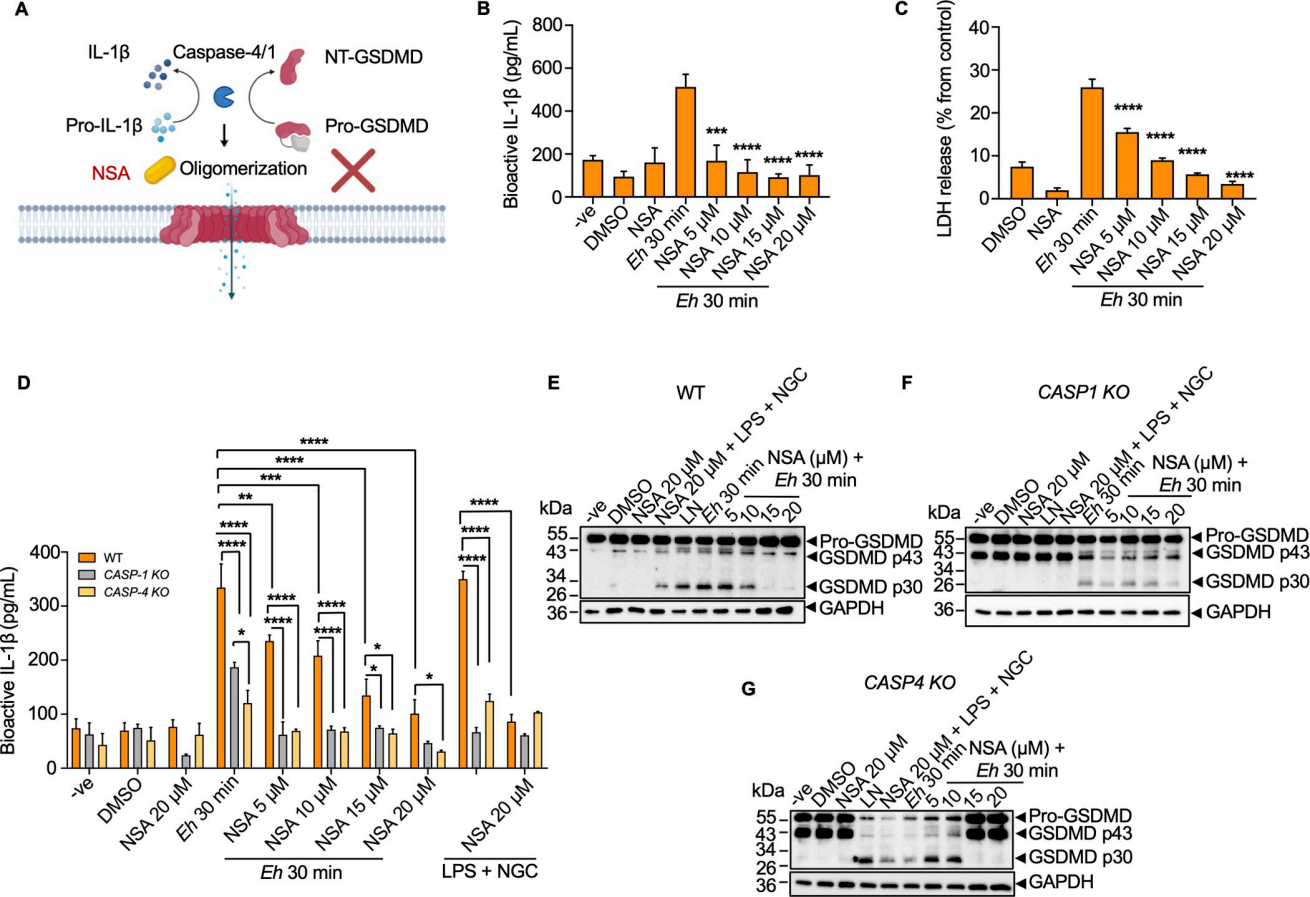
Gasdermin D pores function as gatekeepers of IL-1β release in response to *E*. *histolytica*. (**A**) Necrosulfonamide (NSA) as a direct chemical inhibitor of GSDMD, binds directly to GSDMD, inhibiting oligomerization of GSDMD p30 pore-forming fragment to inhibit pyroptosis. (**B**) NSA was added to macrophages 60 min prior to *Eh* stimulation. Since NSA was prepared in dimethyl sulfoxide (DMSO), DMSO, NSA only (20 μM) were used to detect if DMSO or NSA itself would have any effect on THP-1 cells, and unstimulated cells acted as the negative control. After *Eh* stimulation, cell free supernatant was added to HEK-Blue IL-1β reporter cells to detect bioactive IL-1β using the SEAP assay. (**C**) Pyroptotic pore formation and cell death were assessed through LDH release, cell free supernatants from the same experiments were used to quantify LDH released into the culture supernatant and is shown as a percentage of LDH release compared to non-stimulated cells. (**B, C**) *Eh* treatment only as a positive control and statistical significance was calculated between *Eh* 30 min and various concentration of NSA treatments. (**D**) Cell free supernatant was added to HEK-Blue IL-1β reporter cells to detect bioactive IL-1β using the SEAP assay to detect NSA inhibition in GSDMD pore formation in WT, *CASP1 KO*, *CASP4 KO* macrophage. LPS (50 ng/mL) and NGC (10 μM) stimulation for 60 min acted as the positive control. Statistical significance was calculated between WT and *KO* macrophages and between *CASP1KO* and *CASP4 KO* macrophages at each time point. (**E-G**) Immunoblot analysis was performed for GSDMD p30 cleavage in cell lysates (LYS), and blots were reprobed for GAPDH. WT, CRISPR/Cas9 *CASP1 KO* and CRISPR/Cas9 *CASP4 KO* macrophages were pre-incubated with NSA for 60 min before stimulation with LPS + NGC. Data and immunoblots are representative of six experiments (n = 6) and statistical significance was calculated with Student’s t-test and one-way ANOVA followed by *post hoc* Bonferroni test, (*p < 0.05, **p < 0.01, ***p < 0.001, ****p < 0.0001). Bars represent mean ± SEM.

GSDMD pore formation in hyperactivated macrophages functions as gatekeepers to regulate IL-1β release [20,50]. As GSDMD pores regulate both IL-1β secretion and pyroptosis, we inhibited p30-GSDMD oligomerization [53] with necrosulfonamide (NSA), a chemical antagonist that binds GSDMD to block pores formation and measured IL-1β release and cell death in macrophages stimulated with *Eh* (**Fig 10A**). Increasing concentration of NSA significantly inhibited *Eh*-induced IL-1β secretion and pyroptotic cell death as compared to untreated *Eh*-stimulated macrophages (**Fig 10B–10D**). NSA also significantly inhibited pyroptotic pore formation quantified by IL-1β release in CRISPR/Cas9 *CASP4/1 KO* cells in a dose-dependent manner and in response to LPS + NGC (**Fig 10D**). Under these conditions, cleavage of GSDMD was inhibited with increasing concentrations of NSA in *Eh*-stimulated macrophages as compared to LPS + NGC treated cells implying that both canonical and noncanonical inflammasome activation were affected (**Fig 10E**). In the presence of NSA in *Eh*-stimulated macrophages at 15 and 20 mins, *CASP4 KO* displayed robust downregulation in the cleavage of GSDMD as compared to *CASP1* KO cells (**Fig 10F and 10G**). These data indicate that higher concentrations of NSA correlated with greater decrease in caspase-4 activation, supporting the importance of caspase-4 in cleaving GSDMD in response to *Eh*. These studies also provide a link and potential crosstalk between caspase-4/1 and cleavage of GSDMD that controls pyroptosis.

### Gasdermin D is a proteolytic substrate for caspase-4/1

**Fig 11:**
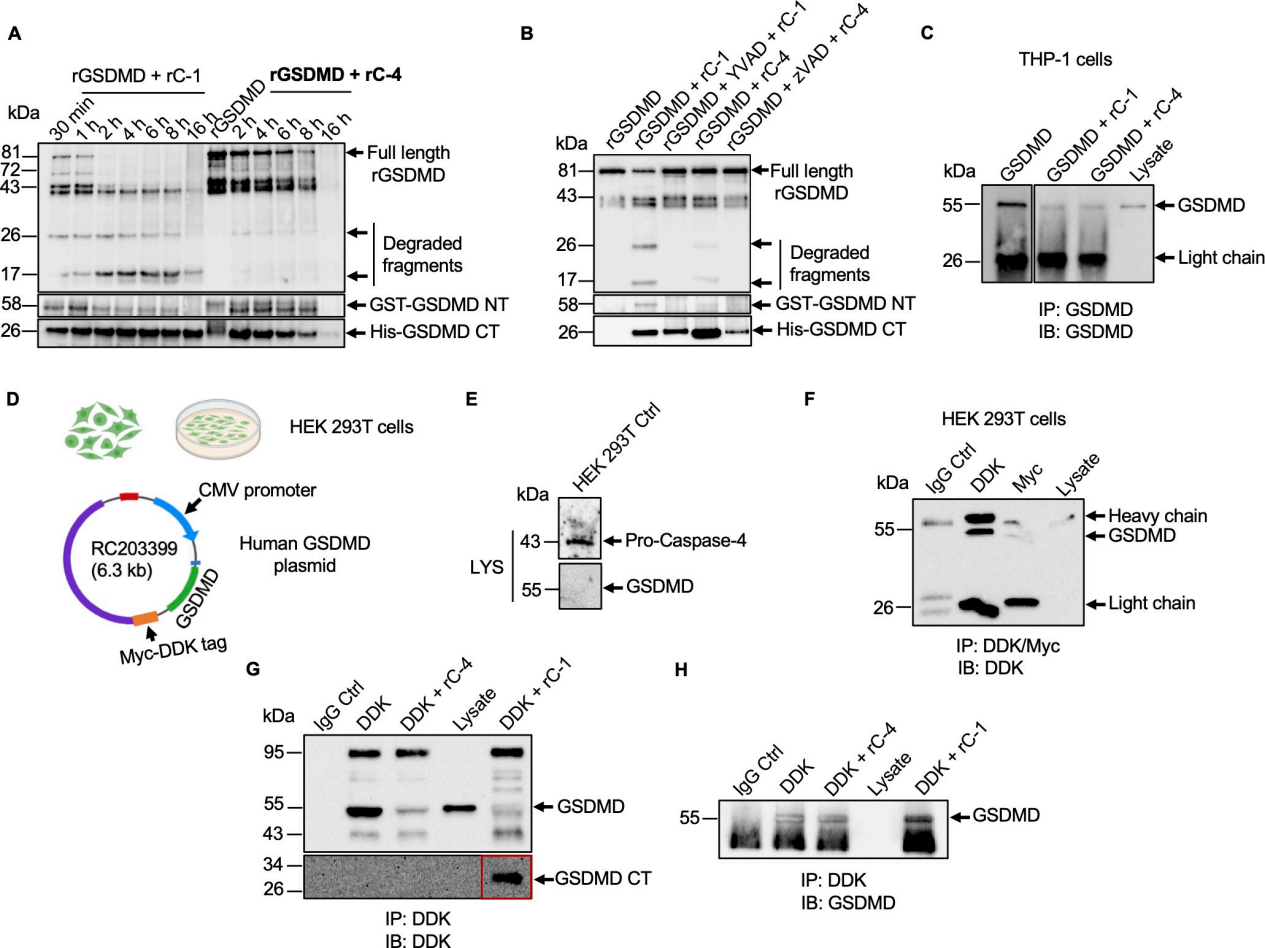
Full length gasdermin D is a proteolytic substrate for active caspase-4/1. (**A**) GST and His-tagged recombinant GSDMD (rGSDMD) was incubated with 1U recombinant caspase-1 (rC-1) and recombinant caspase-4 (rC-4) at 37°C with indicated time points, following the detection by immunoblot analysis. (**B**) rGSDMD were incubated for 15 min at 37°C with active rC-1 and rC-4 in absence or presence of inhibitor Z-VAD-fmk and Z-YVAD-fmk (100 μM, 10 min, room temperature) and rGSDMD cleavage was assessed by western blot with anti-GSDMD, anti-GST and anti-His antibody. (**C**) Macrophages were immunoprecipitated with anti-GSDMD antibody and immunoprecipitants were incubated with active rC-1 and rC-4 for 16 h at 37°C and GSDMD cleavage was assessed by western blotting identified with anti-GSDMD antibody. Direct cell lysate was used as a control (Ctrl). (**D**) C terminal Myc-DDk-tagged human GSDMD plasmid was overexpressed in HEK 293T cells and, (**E**) Basal expression of GSDMD was detected via anti-GSDMD antibody. (**F-H**) HEK 293T cells transfected with Myc-DDk-tagged GSDMD plasmid were immunoprecipitated with anti-DYKDDDDK tag antibody. Immunoprecipitants were incubated with active rC-4 and rC-1 for 16 h at 37°C and GSDMD cleavage was assessed by western blot with anti-DYKDDDDK tag and anti-GSDMD antibody. Immunoblots are representative of at least three independent experiments (n = 3).

To explore mechanistically how *Eh*-induced caspase-4/1 regulates the cleavage of GSDMD, we determined the efficiency of the enzymes to cleave recombinant GSDMD. To do this, GST tagged GSDMD-NT and His-tagged CT human recombinant GSDMD (rGSDMD) were incubated with active recombinant caspase-1 (rC-1) and caspase-4 (rC-4) and the degraded fragments visualized by immunoblots (**Fig 11A**). rC-1 cleaved rGSDMD as early as 30 min whereas, the cleavage with rC-4 was gradually as depicted by the presence of GST-tagged NT-rGSDMD as compared to rC-1 (**Fig 11A**). Incubation with rC-1 and rC-4 at later time points (2–16h) revealed that rC-1 cleaved rGSDMD more rapidly than rC-4 with two prominent degraded cleavage fragments at 26 and 17kDa (**Figs 11A and S5A**). After 16 h enzymatic digest with rC-4, the GST-GSDMD-NT and His-GSDMD-CT were completely degraded whereas, both the GST and His tags were intact in the presence of rC-1. Silver-stained gels of higher sensitivity (**S5B Fig**) revealed that incubation with rC-1 resulted in rapid cleavage of rGSDMD within 5 min with degraded fragments at 58 and 25 kDa that correlated with the GST-GSDMD NT and His-GSDMD CT shown on the immunoblots. rC-4 cleavage of rGSDMD revealed comparable fragments at 58 kDa (NT), 25 kDa (CT) and 17 kDa (arrows). However, when immunoblots were probed with either GST or His antibody, only one cleavage product at 25 kDa was shown with either rC-1 or rC-4, suggesting that both caspase-4/1 cleave GSDMD only at one specific site (**S5C Fig**). Specificity for caspase-4/1 cleavage was shown with the pan-caspase inhibitor Z-VAD-fmk, and the caspase-1-specific inhibitor Z-YVAD-fmk, that completely inhibited the degradation of rGSDMD (**Fig 11B**).

rC-1 and rC-4 also completed degraded native GSDMD that was immunoprecipitated (IP) from macrophage cell lysates incubated for 16 h at 37°C in a similar fashion as the recombinant protein (**Fig 11C**). We also expressed full length GSDMD in human embryonic kidney (HEK) 293T cells containing a Myc-DDK tag (**Fig 11D**) that was used to pull down overexpressed GSDMD. HEK 293T cells do not constitutively express GSDMD (**Fig 11E**) and the IP GSDMD containing the Myc-DDK tag (**Fig 11F**) incubated with active human rC-4 or rC-1 for 16 h was also completely degraded (**Fig 11G**). Shorter incubation times (30 min and 2 h) did not significantly degrade GSDMD (**S5D Fig**). Of interest, rC-1 cleaved DDK tagged GSDMD CT (C-terminus) was detectable in the cell lysate, whereas, it was absent in the presence of rC-4, suggesting that caspase-1 enzymatic activity might be more efficient at cleaving GSDMD. The same blot was reprobed with anti-GSDMD antibody to confirm that GSDMD was efficiently immunoprecipitated from HEK 293T cells (**Fig 11H**).

### Identification of caspase-4 cleavage site on gasdermin D

**Fig 12:**
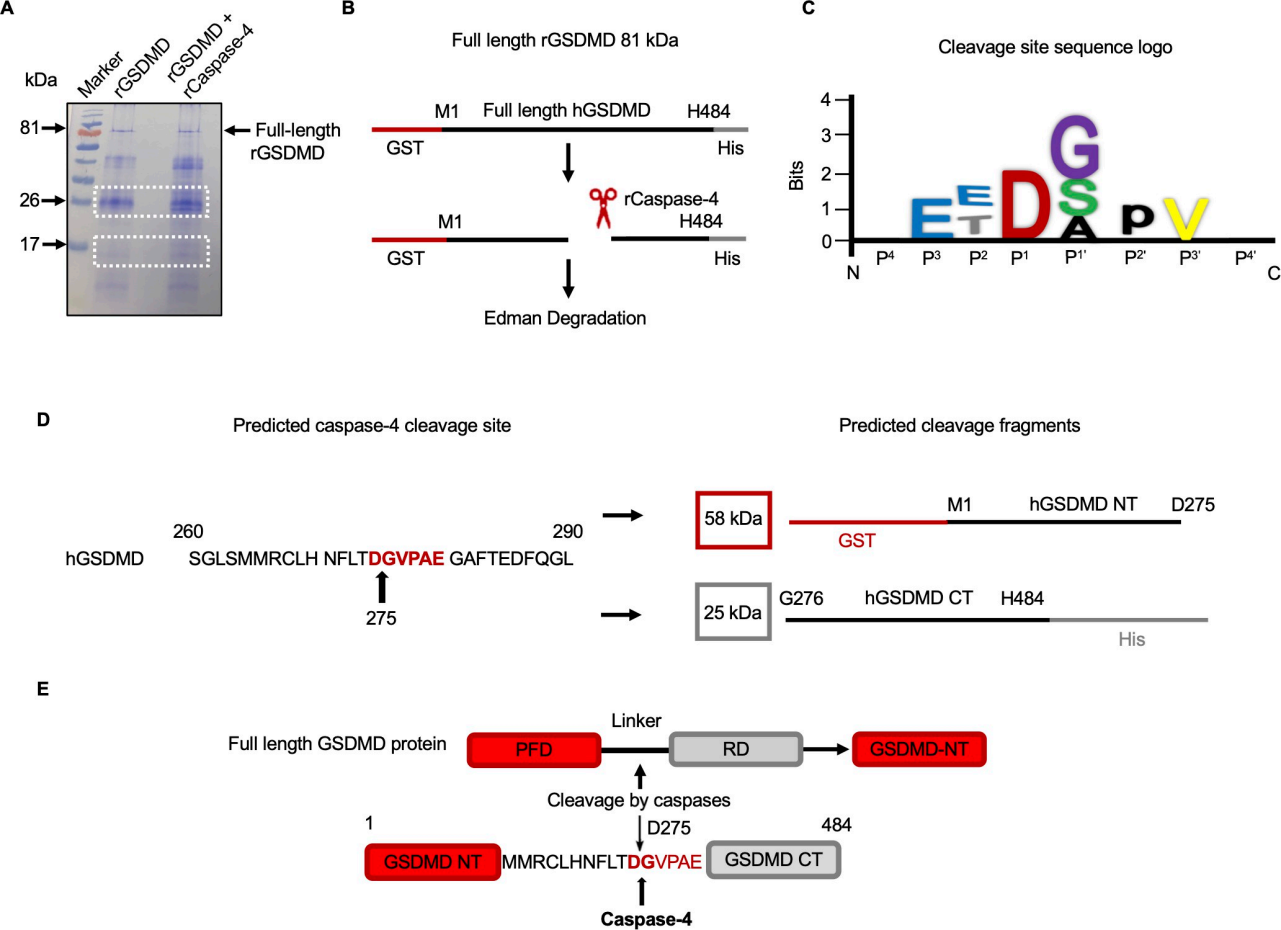
Predicated cleavage site for caspase-4 on GSDMD. The molecular weight of full length rGSDMD is 81 kDa as a GST tag is linked to NT of rGSDMD, whereas CT is tagged by His. (**A**) Cleavage of 5 μg purified recombinant GSDMD with 6U recombinant caspase-4 visualized by Coomassie blue staining of the protein bands that were excised (white dotted box) and sequenced by Edman degradation. Immunoblot is representative of at least three independent experiments (n = 3). (**B**) Schematic representation showed full length rGSDMD with tags. Red line indicated GST tag attached to the NT, while CT was linked by a His tag in grey, and full GSDMD sequence is marked in black. (**C**) Caspase-4 cleavage sequence logo was generated from “MEROPS” (https://www.ebi.ac.uk/merops/) based on the peptidase database. (**D**) After alignment of the calls that we obtained from Edman degradation, the predicated cleavage for caspase-4 on GSDMD marked the same cleavage site as caspase-1 (black arrow). (**E**) NT GST-tagged rGSDMD incubated with active rC-4 for 16 h at 37°C and degraded fragments generated from full length GSDMD were evaluated by immunoblot analysis followed by Coomassie blue staining. Edman degradation analysis of the 26 kDa band after alignment of amino acid calls suggested a cleavage site for caspase-4 on GSDMD at aspartic acid 275 (D275) position.

The caspase-1 cleavage site within the variable linker region of GSDMD is well-studied, however, it is not known where the cleavage site is for caspase-4. To identify the cleavage site of caspase-4 in the linker region of GSDMD, the two major cleaved 26 and 17 kDa fragments (**Fig 12A**) were excised and sequenced by Edman degradation that identified several amino acid calls. Schematic representation of full length rGSDMD (**Fig 12B**) shows the amino acid sequence for the red GST tag on the NT and the grey His tag on the CT. To uncover the cleavage site of the 26 kDa CT fragment, a cleavage specificity preference “logo” for P^4^-P^4’^ position was generated from “MEROPS” (www.ebi.ac.uk), which is a peptidase database based on a cleavage site specificity matrix table (**Fig 12C**). The cleavage site “logo” is a graphic representation of characteristic preference of individual amino acid presented at the P^4^-P^4’^ positions during caspase-4 cleavage. The Single-Letter Amino Acid Code was used to represent the preferable amino acid residues. By multiple sequence alignment analysis, the amino acids were arranged with the GSDMD sequence at asparagine-271 aa position and within these sequences we presumed that the one cleavage site for caspase-4 occurred at Aspartic Acid (D) position 275 (DGVPAE) (**Fig 12D**). By analyzing the cleavage site “logo” and using Expasy (expasy.org) to compute the molecular weight, the predicted cleaved peptide fragments was 58 and 25 kDa, respectively (**Fig 12D**). These data indicate that caspase-4 cleaves GSDMD at a single position that is identical to the caspase-1 cleavage site on human GSDMD (**Fig 12E**).

### Quantitative proteomics analysis of *E. histolytica*-induced hyperactivated macrophage

**Fig 13:**
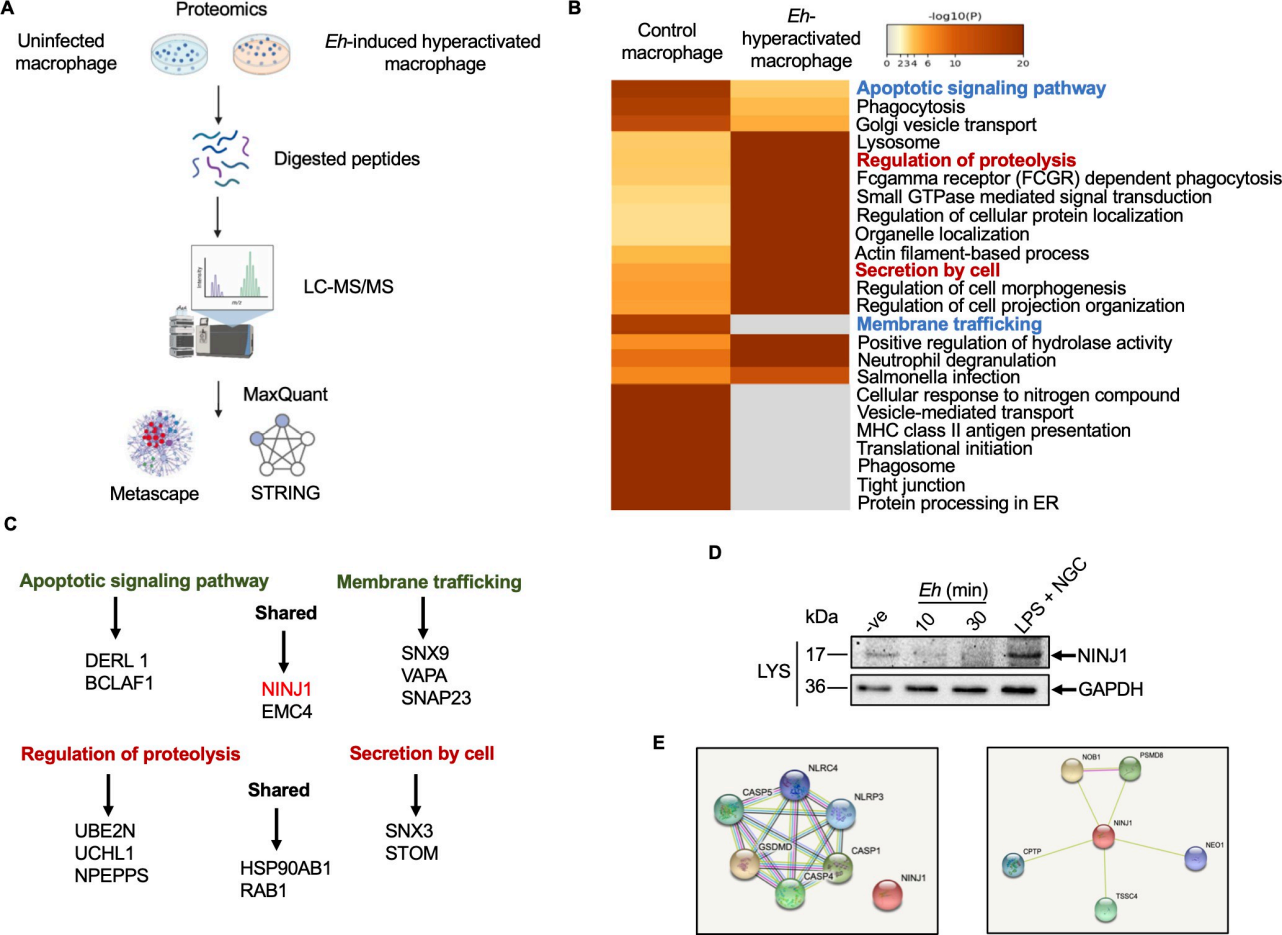
Shotgun proteomics analysis of uninfected macrophages and *E*. *histolytica*-induced hyperactivated macrophages. (**A**) Preparation and workflow for proteomic analysis. (**B**) Metascape analysis of different pathways within control and *Eh*-contacted hyperactivated macrophages. Some upregulated pathways in red and downregulated pathways in blue are what we considered most relevant and interesting. (**C**) Some interesting proteins involved in downregulated and upregulated pathways were characterized, and the common proteins were also indicated. (**D**) Macrophages were incubated with *Eh* (20:1) for 10 and 30 min to detect NINJ1 protein level and blots were reprobed for GAPDH. Immunoblots are representative of at least three independent experiments (n = 3). (**E**) STRING analysis of GSDMD protein-protein interaction, and NINJ1 protein-protein interaction with other top hits proteins were conducted.

**Fig 14:**
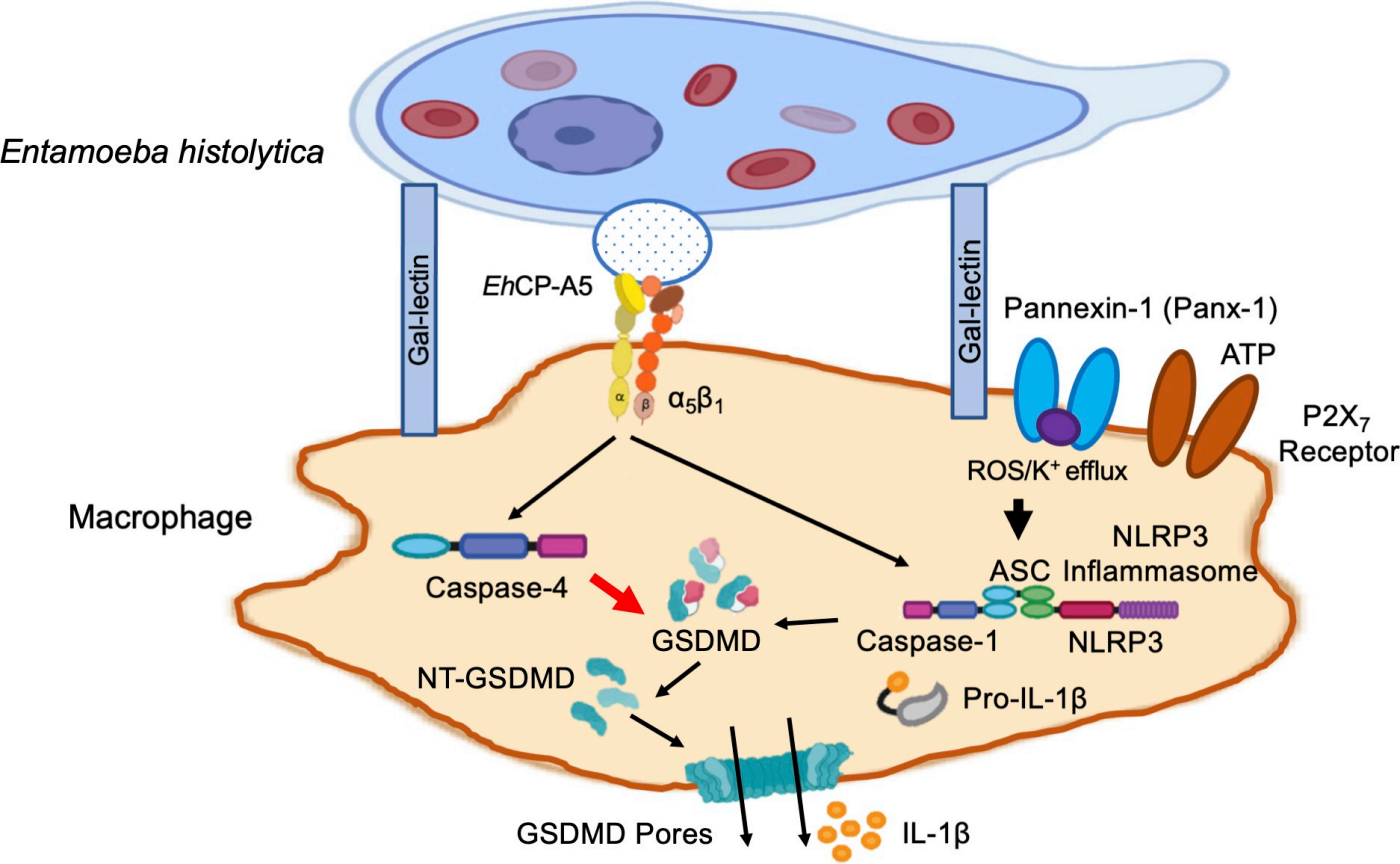
Proposed schematic representation of *E*. *histolytica*-macrophage interaction and induction of caspase-4/1 activation and IL-1β secretion. The activation of caspase is initially triggered by *Eh* in contact with macrophage via the Gal-lectin to Gal/GalNAc residues on the surface of macrophage. *Eh*CP-A5 is highly expressed on the surface of *Eh* and following Gal-lectin binding brings, *Eh*CP-A5 RGD sequences ligate α_5_β_1_ integrin on the macrophage surface to induce the generation of ATP and release through the opening of pannexin-1 channel that subsequently signals back onto the P2X_7_ receptor to activate the NLRP3 inflammasome. Simultaneously, K^+^ efflux and the production of ROS collaborate to activate the NLRP3 inflammasome. The NLRP3 inflammasome in turn activates caspase-1, whereas, the activation of caspase-4 is independent of the inflammasome complex. Whereas both caspase-4/1 acted together to induce the cleavage of GSDMD, caspase-4 played a dominant role in this process. The cleaved GSDMD initiates pore formation allowing bioactive IL-1β release without causing significant cell pyroptosis.

We recently reported a quantitative shotgun proteomic analysis from bone marrow-derived macrophages stimulated with *Eh* for 10 mins [54] with a focus on proteins that regulated the autophagy pathway. Based on this proteomics analysis (**Fig 13A)** database [54], we performed a pathway enrichment and protein networking analysis comparing uninfected macrophages with *Eh*-induced “hyperactivated macrophages” by meta-analysis (metascape.org) [55] that revealed several top downregulated and upregulated pathways [51] (**Fig 13B**). Consistent to our previous findings [51], apoptotic signaling pathway and membrane trafficking were downregulated as indicated in blue, whereas, regulation of proteolysis and secretion by cell were upregulated and marked in red, corresponding to individual downregulated and upregulated proteins that were identified in the pathway enrichment analysis, respectively (**Table 1**). By protein-protein network (STRING-db) [56] analysis, the interactions between upregulated and downregulated pathways from protein-protein enrichment analysis revealed several interesting top hit proteins (**S6A and S6B Fig**). Among the downregulated proteins in regulating membrane trafficking in response to *Eh* were SNAP23, RAB8A and RAB1A. We previously reported that the SNARE vesicle-associated membrane protein (VAMP8) present on mucin granules regulate exocytosis in human goblet cells in the presence of *Eh* [57]. These SNARE complexes are made up of the synaptosome-associated proteins (SNAP) and some syntaxins, as well as many SNARE chaperones to mediate formation of this complex [58]. VAMP8 as the critical vesicle SNARE plays important role in mucin secretion from intestinal goblet cells in response to *Eh* [57], and various SNARE proteins regulate autophagosome formation [59], both ATG7 and SNAP23 were downregulated upon *Eh* stimulation [54]. At present, it is not known what determines whether GSDMD cleavage triggers pyroptosis or hyperactivation. A membrane repair mechanism possibly exists in confronting membrane damage by GSDMD pores, which is capable of rapidly restoring membrane integrity, indicating an important aspect of *Eh*-induced downregulation in SNARE proteins in hyperactivated macrophages. Protein enrichment analysis conducted on approximately 900 proteins that were altered upon *Eh* stimulation shows that comparable amounts of proteins that are upregulated or downregulated in the presence of *Eh* (**S6C Fig**). Among the downregulated proteins (**Fig 13C**), nerve injury-induced protein 1 ninjurin-1 (NINJ1) is of specific interest as it mediates pyroptosis-associated plasma membrane rupture during lytic cell death [60]. The downregulation of the apoptotic pathway in response to *Eh* strongly suggests that “hyperactivated macrophages” switch from apoptosis to pyroptosis [61]. Indeed, macrophages exposed to *Eh* for 10 and 30 min showed marked downregulation of NINJ1 protein expression (**Fig 13D**). These findings suggest that NINJ1 is a mediator of plasma membrane rupture that can release pro-inflammatory cytokines and DAMPs from hyperactivated macrophages in response to *Eh*. Downregulation on NINJ1 in response to *Eh* treatment supports the notion that *Eh* do not cause pyroptosis but triggers GSDMD-regulated hyperactivation in macrophages to elicit a proper pro-inflammatory response. STRING analysis of NINJ1 protein-protein interaction with other pyroptosis-relevant proteins suggested that NINJ1 was not involved in direct interaction with inflammasome-regulated events (**Fig 13E**). Based on the cumulative results of this study, we proposed a model for *E*. *histolytica*-macrophage interaction with activation of primarily caspase-4 (solid red arrow) that interacted with caspase-1 to regulate the cleavage of GSDMD pores for sustained IL-1β secretion (**Fig 14**).

**Table 1:** Identification of the top-hit pathway proteins that were downregulated and upregulated in *E*. *histolytica*-induced hyperactivated macrophages.

Pathway	Protein name	Gene name	Log2
Apoptotic signaling pathway (Downregulated)	Derlin-1	Derl1	-3.408649942
	Serine/threonine-protein kinase OSR1	Oxsr1	-3.018001258
	40S ribosomal protein S27	Rps27	-2.219673101
	Bcl-2-associated transcription factor 1	Bclaf1	-2.171888402
	Eukaryotic translation initiation factor 4 gamma 2	Eif4g2	-1.869596238
	Kinesin-like protein	Kif1b	-1.80450121
	Amyloid beta A4 precursor protein-binding family B member 1-interacting protein	Apbb1ip	-1.80450121
	ER membrane protein complex subunit 4	Emc4	-1.746518942
	Ninjurin-1	Ninj1	-1.6978272
	Protein NDRG1	Ndrg1	-1.693201152
	Apoptosis inhibitor 5	Api5	-1.258977486
Membrane trafficking (Downregulated)	E3 ubiquitin-protein ligase RNF114	Rnf114	-3.223643299
	60S ribosomal protein L29	Rpl29	-3.19588034
	Opioid growth factor receptor	Ogfr	-2.816951839
	Protein transport protein Sec23B	Sec23b	-2.591614209
	Ras-related protein Rab-8A	Rab8a	-2.487297773
	Sorting nexin; Sorting nexin-18	Snx18	-2.472809528
	Unconventional myosin-Ib	Myo1b	-2.319693649
	Paxillin	Pxn	-2.189285452
	Arf-GAP domain and FG repeat-containing protein 1	Agfg1	-1.862706338
	Sorting nexin-9	Snx9	-1.746180117
	Vesicle-associated membrane protein-associated protein A	Vapa	-1.464743289
	Synaptosomal-associated protein 23	Snap23	-1.316635909
Secretion by cell (Upregulated)	Voltage-dependent anion-selective channel protein 3	Vdac3	0.024603367
	ATP synthase subunit gamma	Atp5c1	0.039699918
	Erythrocyte band 7 integral membrane protein	Stom	0.04082231
	Sorting nexin-3	Snx3	0.047538389
	Ras-related protein Rab-1A	Rab1	0.061568849
	Actin-related protein 3	Actr3	0.087598618
	Macrophage-capping protein	Capg	0.143654635
	Heat shock protein HSP 90-beta	Hsp90ab1	0.160274831
Regulation of proteolysis (Upregulated)	Dynactin subunit 1	Dctn1	0.011495639
	Ubiquitin carboxyl-terminal hydrolase isozyme L1	Uchl1	0.015497569
	Eukaryotic translation initiation factor 3 subunit B	Eif3b	0.01564029
	Puromycin-sensitive aminopeptidase	Npepps	0.016353684
	Dipeptidyl peptidase 2	Dpp7	0.019061365
	Ubiquitin-conjugating enzyme E2 N	Ube2n	0.044743821
	Cytosolic non-specific dipeptidase	Cndp2	0.045303168
	UDP-glucose:glycoprotein glucosyltransferase 1	Uggt1	0.058801354
	Succinyl-CoA ligase [ADP-forming] subunit beta, mitochondrial	Sucla2	0.060185765

## Discussion

The focus of this study was to elucidate the molecular mechanisms at the *Eh*-macrophage intercellular junction that activated caspase-4/1 to cleave GSDMD that regulated the secretion of IL-1β in the absence of cell death. We have previously shown that *Eh*-induced caspase-1 activation via the NLRP3 inflammasome was extremely important in the secretion of IL-1β and IL-18 [10]; however, the molecular events that governed the activation of caspase-4 and its cross talk with caspase-1 to regulate IL-1β secretion via GSDMD pores was not known. Here we reveal that *Eh*-induced activation of caspase-4 followed a similar pattern as caspase-1 required a priming signal to upregulate pro-caspase-4 mRNA and protein. Intriguingly, we found that following Gal-lectin binding, ligation of *Eh*CP-A5 RGD sequence to macrophage α_5_β_1_ integrin was an essential trigger to activate caspase-4 in the absence of the NLRP3 inflammasome or any inflammasome components. With the use of CRISPR/Cas9 *CASP4/1 KO* cells, we uncovered that GSDMD was a key downstream substrate for both caspases to regulate pro-inflammatory cytokine secretion and/or pyroptotic cell death. By Edman degradation we identified that the GSDMD enzymatic cleavage site for caspase-4 was identical to caspase-1, however caspase-4 was indispensable in cleaving GSDMD to mediate pore formation that regulated sustained IL-1β release in response to *Eh*. Remarkably, *Eh*-induced high output IL-1β secretion from caspase-4-mediated GSDMD pores was not due to cell pyroptosis, but rather, the parasite seems to program macrophages to reach a “hyperactivated” state for sustained secretion of IL-1β. This was in distinct contrast to LPS + NGC stimulated cells that induced robust expression of caspase-1, high output IL-1β secretion and extensive pyroptotic cell death (~ 80%). Thus, the findings of this study have uncovered a new role for *Eh*-induced activation of caspase-4 that regulated measured GSDMD pore formation and pro-inflammatory secretion in the absence of cell death that may play a role in disease pathogenesis and innate host defence.

A surprising finding was that GSDMD-dependent IL-1β secretion following contact with *Eh* occurred in the absence of significant cell death even after 60 min interaction. HEK IL-1β reporter cells were used to quantify IL-1β release, as it was specific in measuring bioactive IL-1β through the generation of SEAP and the assay was consistency between experiments [6]. More importantly, even though both caspase-4/1 cleaved GSDMD in response to *Eh*, we assumed there would be a temporal order for these caspases to execute their functions and therefore explored which caspase cleaved GSDMD more efficiently/rapidly to regulate IL-1β release. Although both caspase-4/1 were required to orchestrate a competent pro-inflammatory response towards *Eh*, our data suggest that caspase-1 played a dominant role in processing pro-IL-1β into its bioactive form, whereas caspase-4 played a principal indispensable role in cleaving GSDMD for pore formation that regulated IL-1β release (**Fig 7**). These findings are noteworthy, as prior to this study, it was not known if caspase-4 played a dominant role in pore-forming activity during protozoan infections and there were no reports on the caspase-4 cleavage site on GSDMD. By applying GSDMD antagonist to macrophages, an intense reduction in IL-1β secretion and LDH release was detected, especially comparing *Eh* treatment to the positive control, LPS + NGC. These findings suggest that pharmacologically inhibiting GSDMD may be clinically efficacious for treating inflammatory diseases such as familial cryopyrin-associated periodic syndromes, autoimmune conditions and gastrointestinal diseases [62]. Despite the importance of studying the mechanisms that regulate GSDMD pore-forming activity, it is also crucial to reveal the mechanisms that control auto-inhibition, prior to activation and processing of GSDMD. Structural studies have revealed that the crystal structures of active caspases-1/4/11 form a complex with GSDMD-CT [63]. Hence, GSDMD CT functions would not only auto-inhibit GSDMD-NT but might serve as the platform to trigger inflammatory caspases recruitment and GSDMD cleavage.

Although cytosolic LPS and some gram-negative bacteria can trigger the activation of caspase-4 [34,64], we discovered that *Eh*, as an extracellular parasite can induce outside-in signaling in macrophages to activate caspase-4. Intriguingly we found that *Eh* coupling with macrophages via the Gal-lectin and *Eh*CP-A5 to α_5_β_1_ integrin [5,6] resulted in pannexin-1 channel-regulated ATP release that subsequently signals back through ATP-gated P2X_7_ receptors to deliver a co-stimulatory/second signal to trigger the activation of both the NLRP3 inflammasome and noncanonical caspase-4. *In vitro* cleavage assays and Edman sequencing confirmed that the caspase-4 cleavage site on GSDMD was the same as caspase-1 that cleaved aspartic acid at position D275 (26 kDa fragments). GSDMD functions as the gatekeeper to release IL-1β and mediate cell pyroptosis. If few GSDMD pores are generated, the cell might react by initiating compensatory mechanisms to recover its volume. Alternatively, if the number of GSDMD pores patches to cell membrane exceeds the recovery capability of the cell, cell volume in turn increases. Consequently, the opening of GSDMD pore breaks the normal permeability barrier of the plasma membrane, resulting in membrane disruption, leading to pyroptotic cell death. Based on the progressive (temporal) cleavage of GSDMD induced by *Eh* in contact with macrophages that was remarkably similar to what was observed in *CASP1 KO* cells (**Fig 6A and 6B**) with higher IL-1β secretion up to 60 mins in the absence of cells death (**Fig 7C and 7D**), our data suggests that caspase-4 played a dominant role in generating sufficient GSDMD pores that allowed the cells to initiate compensatory mechanisms for its survival.

Pyroptosis is not the only means by which IL-1β is secreted from cells. As shown in this study, GSDMD-dependent IL-1β release triggered by *Eh* was not due to massive membrane rupture and significant pyroptosis, but instead, stimulated macrophages to reach a “hyperactivated stage”. This was somewhat surprising as the use of lytic factors to destroy host cells for nutrient acquisition and immune evasion is a common feature of many invasive pathogens. This could be the typical scenario with *Eh*, or from a broader perspective, extracellular parasites that depends on outside-in signaling not to cause phagocytes death. Hence, it is of critical importance to determine what triggers cell hyperactivation and what are the consequences and significance of this “hyperactivation status”. Unravelling the underlying mechanisms on how *Eh* manipulate macrophages to be “hyperactivated” might shed light on new strategies to alleviate amebiasis. Additionally, as plasma membrane rupture (PMR) was widely believed to occur spontaneously and passively after cell pyroptosis, it was interesting to demonstrate a requirement for NINJ1 to trigger pyroptosis-related and -unrelated PMR in mouse bone marrow derived macrophages [60]. In future studies, it will be interesting to dissect the relationship between GSDMD-triggered IL-1β secretion and NINJ1-dependent PMR in response to *Eh*. Our data provide promising evidence that the release of IL-1β form macrophages is independent of PMR and probably occurs via the approximately 10–15 nm GSDMD pores (IL-1 family cytokines have a diameter of 4.5 nm) [16,18]. However, what defines whether GSDMD cleavage causes pyroptosis or hyperactivation in macrophages remains unclear. There is emerging evidence that indicate a membrane repair response is triggered to combat membrane damage by GSDMD pores, and cells can sense membrane disruption by an instant boost of intracellular Ca^2+^ to trigger membrane repair by replenishing the endosomal sorting complexes required for transport (ESCRT) [65]. Proteomic analysis on *Eh*-induced hyperactivated macrophages revealed downregulation in membrane trafficking, suggesting *Eh* hijacks this process and elicit a vigorous inflammatory response. Cell survival and membrane disruption potentially reflects the competition between how severe and how rapidly membrane is disintegrated, versus the efficiency of the repair process, which is possibly relied upon by how much GSDMD pore are formed. Various intensity of inflammatory stimuli and degree of caspase expression and activation can be essential determinants to establish cell pyroptosis or hyperactivation. It is worth noting that CRISPR/Cas9 *GSDMD KO* cells still release detectable levels of IL-1β in response to *Eh* (**Fig 9**) thus, a more in depth understanding of unconventional cytokine and the regulation of cell pyroptosis should be explored.

There is still much to be learned in innate defenses that underpin pro-inflammatory responses initiated by *Eh* via the canonical inflammasome-dependent and -independent pathways. We still do not understand how the host recognize and elicits an adequate level of response by assessing the level of threat caused by *Eh*. In this regard, our study advances plausible mechanisms of how inflammatory caspase-4/1 are activated through outside-in contact-dependent signaling at the synapse between *Eh* and host immune cells. A major finding was that *Eh* triggered a “hyperactivated macrophage” state for high output IL-1β production in the absence of extensive cell pyroptosis despite efficient cleavage of GSDMD by activated caspase-4/1. These findings unravel new concepts on how *Eh*-induced inflammatory caspases can shape the magnitude of host pro-inflammatory responses that can play a role in disease pathogenesis and innate host defense.

## Materials and methods

### Cultivation, harvesting of *E*. *histolytica*

*E*. *histolytica* virulent strain, HM-1:IMSS were grown axenically in TYI-S-3 medium supplemented with 100 U/mL penicillin and 100 μg/mL streptomycin sulfate at 37°C in sealed 15 mL borosilicate glass tubes [66]. In order to maintain virulence, trophozoites are regularly passed through gerbil livers [67]. *E*. *histolytica* were harvested after 72 h of log-phase growth by placing on ice for 5 min and then centrifuged at 200 ×*g* for 5 min at 4°C. After centrifugation, *Eh* were resuspended in serum-free RPMI to count and prepared a final cell suspension of 1×10^6^ *Eh*/mL. *Eh*CP-A5 deficient *Eh* were a generous gift from Dr. David Mirelman (Weizmann Institute of Science, Rehovot, Israel) and cultured similarly.

### Cell preparation and stimulation

THP-1 human monocytic cells (ATCC, Manassas, VA) were maintained in RPMI-1640 supplemented with 10% (vol/vol) heat-inactivated fetal bovine serum (FBS), 10 mM HEPES, 50 μM β-mercaptoethanol, and 100 U/mL penicillin and 100 μg/mL streptomycin sulfate in a humidified cell culture incubator with 5% CO_2_. For experiments, 8×10^5^ THP-1 cells per well were seeded onto 12-well tissue culture plates in complete RPMI containing Phorbol 12-myristate 13-acetate (PMA) (50 ng/mL) in complete RPMI medium overnight to induce differentiation into macrophages the night before experiment. For *Eh* experiments, cells were stimulated with WT *Eh*, *Eh*CP-A5^-^ in a final volume of 400 μL serum-free RPMI-1640 for indicated incubation time with *Eh* to THP-1 macrophage at 1:20 ratio at 37°C. For inhibitor studies, PMA-differentiated THP-1 macrophages were pre-treated with inhibitors for indicated concentrations and time at 37°C prior to *Eh* stimulation, unless otherwise stated. Oxidized ATP (A6779), ultra-pure LPS from *E*. *coli* 0111: B4 (L3012), PMA, and nigericin (N7143), ATP (A7699), glucose, and D-galactose were obtained from Sigma-Aldrich (St Louis, MO). Z-VAD (OMe)-FMK and Z-YVAD (Ome)-FMK were from Enzo Life Sciences. Necrosulfonamide (NSA) (AG-CR1-3705, Adipogen International), purified native Gal-lectin was a gift from Dr. David Lyerly (Techlab, Blacksburg, Virginia), Carbenoxolone (C 4790, Sigma-Aldrich), Probenecid (P 8761, Sigma-Aldrich). After *Eh* treatment, THP-1 supernatants from three wells were pooled and centrifuged at 4°C for 5 min at 2000 ×*g*. Pelleted debris was discarded and supernatants were concentrated by trichloroacetic acid (TCA) precipitation. Plates were washed with cold PBS and lysis buffer (1% Triton X-100, 20mM Tris, 100 mM NaCl, 1 mM EDTA, 200 mM orthovanadate, sodium fluoride, 0.1% sodium dodecyl sulfate (SDS), phenylmethanesulfonyl fluoride (PMSF), leupeptin, aprotinin, and protease inhibitor cocktail) was added to lyse the cells. Protein concentrations were determined by the bicinchoninic acid protein assay kit, using bovine serum albumin as a standard (Thermo Scientific, Catalog No. CAPI23225).

### CRISPR/Cas9 KO gene editing of THP-1 cells

Caspase-1 and caspase-4 CRISPR/Cas9 *KO* THP-1 cells were a gift from Dr. V. Hornung (Institute of Molecular Medicine, University Hospital, University of Bonn, Germany). To generate this cell, CMV-mCherry-CAS9 expression cassette encoded plasmid and a gRNA under the U6 promoter was used. The CRISPR target regions were: ATTGACTCCGTTATTCCGAA**AGG** (Caspase-1) and GCTCATCCGAATATGGAGGC**TGG** (Caspase-4), PAM regions in bold. All CRISPR KO THP-1 cells were cultured in complete RPMI media as described above.

ASC def THP-1 cells were purchased from InvivoGen (thp-dasc). These cells were originally obtained from THP-1 human monocytic cells with no expression of ASC, but express native levels of NLRP3 and pro-caspase-1. CRISPR/Cas9 *NLRP3 KO* THP-1 cells were a generous gift from Dr. D. Muruve (Department of Immunology, University of Calgary, Canada). They used the gRNA CTGCAAGCTGGCCAGGTACC**TGG** and TGTCATAGCCCCGTAATCAA**CGG**. CRISPR Cas9 *GSDMD KO* THP-1 cells were a generous gift from Dr. Bachovchin (Memorial Sloan Kettering Cancer Center, New York, USA). The sgRNA used was TGAGTGTGGACCC-TAACACC.

### Immunoblot

Equal amounts of supernatants and lysates were resuspended in Laemmli buffer, boiled for 5 min and were loaded and resolved on SDS-polyacrylamide gel electrophoresis and transferred to nitrocellulose membrane followed by blocking in 5% skim milk, incubated overnight at 4°C in indicated primary antibodies, and incubated with appropriate secondary horse radish peroxidase-conjugated antibodies. Protein bands were detected with either SuperSignal Chemiluminescence Reagents (Pierce, Biotechnology) or ChemiLucent ECL detection (EMD Millipore). To detect the loading control protein (GAPDH), membranes were treated with stripping solution (25 mM Glycine, 1% SDS, pH = 2.0) for 15 min. After properly washed, 5% skim milk was used to block the membrane and then incubated with indicated primary antibody. Primary antibodies used were: anti-IL-1β (H-153) (7884, Santa Cruz), anti-caspase-1 human (622, Santa Cruz), anti-caspase-4 human (M029-3, MBL International Corporation), anti-GSDMD (96458, Cell Signaling), anti-GAPDH (Millipore, Sigma), anti-GST (086, Delta Biolabs), anti-His tag (A00186-100, GenScript), anti-DYKDDDDK tag antibody (anti-FLAG M2) (Sigma-Aldrich), anti-Myc (098, Delta Biolabs) and anti-NINJ1 (12H6.1, Sigma-Aldrich). For immunoprecipitation blots, secondary antibodies used were: Goat Anti-Mouse light chain Antibody (Bethyl Laboratories, Inc.), HRP conjugate (Sigma-Aldrich) and HRP-conjugated Goat Anti-Mouse IgG Heavy Chain (abclonal).

### Human gasdermin D plasmid transfection to HEK 293T and *in vitro* caspase-cleavage assay

HEK 293T cells were maintained in Dulbecco’s modified Eagle’s medium (DMEM) supplemented with 10% FBS and antibiotics. HEK 293T cells (1.0 × 10^6^/well) were transiently transfected using jetPRIME Polyplus transfection reagent with human pCMV6-Gsdmd (Origene) plasmid, encoding the full-length GSDMD containing both a DYKDDDDK (FLAG) tag and a Myc tag on the C-terminal of the pLenti-C-Myc-DDK Lentiviral Gene Expression Vector. After 24 h, cells were lysed, collected and immunoprecipitated with anti-DYKDDDDK (FLAG) tag antibody followed by protein A/G PLUS-Agarose (Santa Cruz) conjugation at 4°C. Coupled protein bound beads were resuspended into caspase cleavage buffer (50 mM HEPES, pH 7.2, 50 mM NaCl, 0.1% CHAPS, 10 mM EDTA, 5% glycerol and 10 mM DTT). 4U active recombinant human caspase-4 (ENZO) and caspase-1 (ENZO) were incubated with the isolated proteins for 16 h at 37°C. After 16 h of incubation, samples were examined by immunoblot analysis with both anti-GSDMD (cell signaling) and anti-DYKDDDDK (FLAG) tag antibody.

### Immunoprecipitation

For immunoprecipitation, anti-GSDMD (A305-736A-M, Bethyl Laboratories, Inc.) was used to pull down proteins from cell lysate. Immunoprecipitation of HEK 293T cells was described as above. Cells were lysed in lysis buffer (50 mM Tris-Cl pH 8.0, 150 mM NaCl, 1% NP40, 5 mM EDTA, 0.1% CHAPS) supplemented with protease inhibitor cocktail. 200 μg lysates were incubated with the relevant antibody at 4°C before adding protein A/G PLUS-Agarose (Santa Cruz) for 2 h. Beads were washed three times with the same buffer and bound proteins were eluted with Laemmli buffer by boiling for 5 min. Post immunoprecipitation, complexes of beads-protein were incubated with recombinant caspase-1 (8U, ENZO) and recombinant caspase-4 (8U, ENZO) 16 h at 37°C. Protein complexes were washed three times with lysis buffer and incubated at 95°C for 5 min and resolved by immunoblotting.

### Silver staining and immunoblot analysis of total proteins by *in vitro* caspase-cleavage assay

Human recombinant GSDMD was obtained from Origene, and recombinant caspase-1 and recombinant caspase-4 were purchased from Enzo life sciences. For visualization of secreted proteins blots were silver stained as per the manufacturer’s protocol (Pierce Silver Stain kit, # 24612). For inhibition studies, 100 μM pan caspase inhibitor Z-VAD-FMK and caspase-1 Z-YVAD-FMK was added for 10 min at room temperature prior to recombinant caspase-1 and caspase-4 incubation. After the indicated incubation times, samples were examined by western blot with anti GSDMD, anti-GST and anti-His antibodies.

### IL-1β assay and HEK-Blue reporter cells

Following treatment of THP-1 macrophages, supernatants were kept on ice for immediate processing or frozen at -80°C. In the following morning, HEK-Blue IL-1β cells were seeded onto a 96-well plate at 8 x 10^5^ cells/mL with a total of 100 μL in each well. Next, 100 μL of supernatants from THP-1 macrophages were added undiluted into each well containing HEK-Blue IL-1β cells overnight at 37°C in an incubator with 5% CO_2_. A standard curve using human recombinant IL-1β (200-01B, Peprotech) was made with serial dilutions (100 to 0.01 ng/μL). A total of three replicates were performed for each treatment condition. The following day, 100 μL of supernatant was transferred into a black 96-well plate. Next, 100 μL of the QUANTI-Blue solution containing reagent (rep-qbla, InvivoGen) and QUANTI-Blue buffer (rep-qblb, InvivoGen) were added into each well. The QUANTI-Blue solution is initially pink and eventually turns into blue with incubation, as indicative of SEAP levels. Bioactive IL-1β levels gradually increased and the intensity of the color reaction is proportional to the amount of IL-1β in the supernatant from stimulated THP-1 macrophages. The plate was incubated at 37°C for 90 min and the SEAP levels were assessed using a spectrophotometer at 655 nm.

### Cytotoxicity assay (LDH release)

Lactate dehydrogenase (LDH) released into extracellular media from macrophage culture was measured with the Promega CytoTox-ONE homogeneous membrane integrity assay (G7890, Fisher Scientific) following the instruction from the manufacturer. Relative LDH release was calculated using the equation: LDH (% release) = % of (LDH released from stimulation-background) / (maximum LDH released-background). This was performed to calculate percent cell death relative to a complete cell lysis control and the LDH (% released from control) was calculated using the LDH (% release) subtracted from non-stimulated cells.

### Edman protein sequencing

Active recombinant caspase-4 were incubated with GST-tagged human recombinant GSDMD for 2 h at 37°C. After 2 h of incubation, samples were resolved on SDS-PAGE gel and transferred to polyvinylidene difluoride (PVDF) membrane. Coomassie blue staining was used to visualize the degraded fragments. After detaining the membrane, each fragment band was cut and sent for Edman sequencing at Tufts University core facility.

### Proteomic data and bioinformatics analysis

Uninfected macrophages and *E*. *histolytica*-induced hyperactivated macrophages were used for shotgun proteomics analysis. Detailed preparation and data collection process was as described previously [54]. MaxQuant [68] software was used for a peptide-spectrum match at a 1% false discovery rate (FDR), log2 of value was implemented to interpret changes in protein abundance.

### mRNA expression analysis by real-time qPCR

Total RNA was extracted from snap-frozen tissue using E.Z.N.A. Total RNA Kit (Omega BioTEK) by following Trizol reagent protocol (Invitrogen; Life Technologies, Burlington, ON) per manufacturer’s instructions. The purity and yield of the RNA was detected by the ratio of absorbance at 260/280 nm (NanoDrop, Thermo Scientific). qScript cDNA synthesis kit was used to prepare complementary DNA (cDNA). Rotor Gene 3000 real-time PCR system (Corbett Research) was used for mRNA expression analysis. Each reaction mixture contained 1:10 dilution of prepared cDNA, SYBR Green PCR Master Mix (Qiagen) and 1 μM of primers (F + R). Results were analyzed using the 2^−ΔΔCT^ methods and expressed as fold changes relative to housekeeping genes. The primer sequences and conditions used are listed below: Human GAPDH, F: GGATT TGGTCGTATTGGG, R: GGAAGATGGTGATGGGATT; Human caspase-4, F: AAGAGAA-GCAACGTATGGCAGGAC, R: GGACAAAGCTTGAGGGCATCTGTA

### Statistics

All experiments shown are representative of at least three independent experiments. Densitometry analysis was performed by the Image Lab software. GraphPad Prism 8 (Graph-Pad Software, San Diego, CA) was used for statistical analysis. Statistical significance between two groups was done by Student’s t test and comparison between two or more groups were done by one-way analysis of variance (ANOVA), followed by *post hoc* Bonferroni test. Statistical significance was considered at *p* < 0.05. Results are displayed as mean ± standard error of the mean (SEM). ImageLab Software Version 6.0 was used for western blot analysis and to determine densitometric values from three independent experiments. Results were reported as mean ± SEM.

## Supporting information

S1 Fig *E*. *histolytica* activates both caspase-4 and caspase-1 in a dose dependent fashion. (**A**) Macrophages were incubated with increasing *Eh*-macrophage ratios using LPS + NGC as a positive control. Unstimulated macrophages were used as a negative control. Cell supernatant (SN) was TCA precipitated and equal amount of proteins was resolved on SDS-PAGE following the investigation of activated caspase-4 and caspase-1 that was secreted into cell supernatant. (**B**) Cell supernatant from macrophages was added to HEK-Blue reporter cells to detect bioactive IL-1β via the SEAP assay macrophages that were incubated with *Eh* for increasing *Eh*-macrophage ratios. (**C**) Cell death was also determined by LDH released into cell culture and is shown as a percentage of LDH release compared to non-stimulated cells. Data and immunoblots are representative of at least three independent experiments (n = 3) and statistical significance was calculated with an ANOVA and Bonferroni’s *post-hoc* test between each *Eh*-macrophage ratio and positive control treatment, (**p < 0.01, ****p < 0.0001). Bars represent mean ± SEM.
(TIFF)

S2 Fig Exogenous ATP does not restore inflammasome activation in response to *Eh*CP-A5^-^*Eh*, but slightly increased caspase-4 activation in the absence of caspase-1. (**A, B**) Both WT and CRISPR/Cas9 *CASP-1 KO* macrophages were incubated with 5 mM exogenous ATP from 30 min to 4 h. Restored activation of caspase-4 was detected in *CASP1 KO* cells stimulated with ATP for 4 h (red box). (**C, D**) Immunoblot analysis was performed for active caspase-4 and caspase-1 products and IL-1β assay in HEK-Blue reporter cells from macrophages stimulated for 60 or 90 min with WT *Eh* and *Eh*CP-A5^−^*Eh*, respectively. Statistical significance was calculated between each treatment at the same time points (**E, F**) Exogenous ATP slightly restored caspase-4 activation to rescue IL-1β secretion in the absence of caspase-1 in response to *Eh*CP-A5^-^*Eh*. Cell supernatant was TCA precipitated and equal amount of supernatants (SN) was loaded onto SDS-PAGE and immunoblot analysis was performed for caspase-4, caspase-1 and IL-1β. IL-1β assay in HEK-Blue reporter cells from both WT and *CASP1KO* macrophages was quantified and statistical significance was calculated between each treatment under the same cell types. Data and immunoblots are representative of at least three independent experiments (n = 3) and statistical significance was calculated with one-way ANOVA, followed by Bonferroni’s *post-hoc* test, (*p < 0.05, ****p < 0.0001, ns: not significant). Bars represent mean ± SEM.
(TIFF)

S3 Fig Gasdermin D cleavage was increased with *Eh*CP-A5 deficient *Eh*. (**A**) WT THP-1 macrophages were incubated with *Eh*CP-A5^-^*Eh* and WT *Eh* for 60 and 90 min, respectively. Cells were washed and lysed and equal amount of cell lysates was loaded onto SDS-PAGE and immunoblot analysis was conducted to investigate GSDMD cleavage. (**B**) GSDMD p30 fragment presented in the cell lysate was confirmed with densitometry quantification. (**C**) IL-1β secretion was quantified in HEK-Blue reporter cells. Data and immunoblots are representative of at least three independent experiments (n = 3) and statistical significance was calculated with one-way ANOVA and *post hoc* Bonferroni test between WT *Eh* and *Eh*CP-A5^−^*Eh*, (**p < 0.01, ***p < 0.001, ****p < 0.0001). Bars represent mean ± SEM.
(TIFF)

S4 Fig Gasdermin D cleavage depends on caspase-4 activation that requires pannexin-1 channels. (**A, B**) Macrophages were pretreated with carbenoxolone (CBX), a connexin/pannexin channel dual inhibitor, or with the pannexin channel inhibitor probenecid (PB) for 30 min prior to *Eh* stimulation. Cells were washed and lysed and equal amount of cell lysates was loaded onto SDS-PAGE and immunoblot analysis was performed for GSDMD p30 cleaved fragment. (**C, D**) Macrophages were pre-incubated with the pan-caspase inhibitor Z-VAD-fmk (100 μM) and caspase-1 specific inhibitor Z-YVAD-fmk (100 μM) for 45 min followed by stimulation with *Eh* (20:1 ratio) for 30 min. The caspase inhibitors were also used alone to tests whether it had an effect on GSDMD cleavage. GSDMD cleaved fragments in the cell lysate were assessed via immunoblotting. Western blots and densitometric analysis are representatives of at least three independent experiments (n = 3). Statistical significance was calculated with one-way ANOVA and *post hoc* Bonferroni test between *Eh* stimulation for 30 min and with the addition of inhibitors, (****p < 0.0001). Bars represent mean ± SEM.
(TIFF)

S5 Fig GSDMD protein is a proteolytic substrate for active caspase-4 and caspase-1. (**A**) rGSDMD was incubated with active rC-1 and rC-4 for the same amount of time (2 h, 4 h and 16 h) and immunoblot analysis was conducted to assess the degraded fragments of GSDMD. (**B**) Cleavage of rGSDMD by active rC-1 and rC-4 was conducted at 37°C for indicated incubation time, following by silver staining. rGSDMD was incubated at 37°C for 2 h in the absence of recombinant caspases to investigate if any autoproteolysis exists. (**C**) Full blots of the cleavage assay conducted on rGSDMD with rC-1 and rC-4 and detected by anti-GST and anti-His antibodies. (**D**) C terminal Myc-DDk-tagged human GSDMD plasmid was overexpressed in HEK 293T cells and immunoprecipitated with anti-DYKDDDDK antibody. Immunoprecipitants were incubated at 37°C with active rC-4 for various time points (30 min, 2 h and 16 h) and GSDMD cleavage was assessed by western blot with anti-DYKDDDDK and anti-GSDMD antibody. Direct cell lysate was used as a control. Immunoblots are representative of at least three separate experiments (n = 3).
(TIFF)

S6 Fig Protein-protein interaction enrichment analysis on downregulated and upregulated pathways following *E*. *histolytica* contact with macrophage. Based on the proteomics analysis in *Eh*-induced hyperactivated macrophages, a protein-protein interaction assay was conducted by STRING protein-protein interaction analysis. (**A**) Shown is the network of enriched terms within the downregulated pathway (Apoptotic signaling pathway and membrane trafficking), where connections that share the same cluster typically interact to each other. (**B**) Network demonstrating the interactions between downregulated pathway and upregulated pathway (Regulation of proteolysis and secretion by cell) in hyperactivated macrophages by STRING analysis. (**C**) Volcano plot of proteins from both downregulated and upregulated pathways in *Eh*-induced hyperactivated macrophages with log2 interpretation.
(TIFF)
