# Supplementary figures and images for: The colonic pathogen Entamoeba histolytica activates caspase-4/1 that cleaves the pore-forming protein gasdermin D to regulate IL-1β secretion
Source: PLoS Pathog. 2022 Mar 18;18(3):e1010415. doi: 10.1371/journal.ppat.1010415 (PMC8967020; doi:10.1371/journal.ppat.1010415)

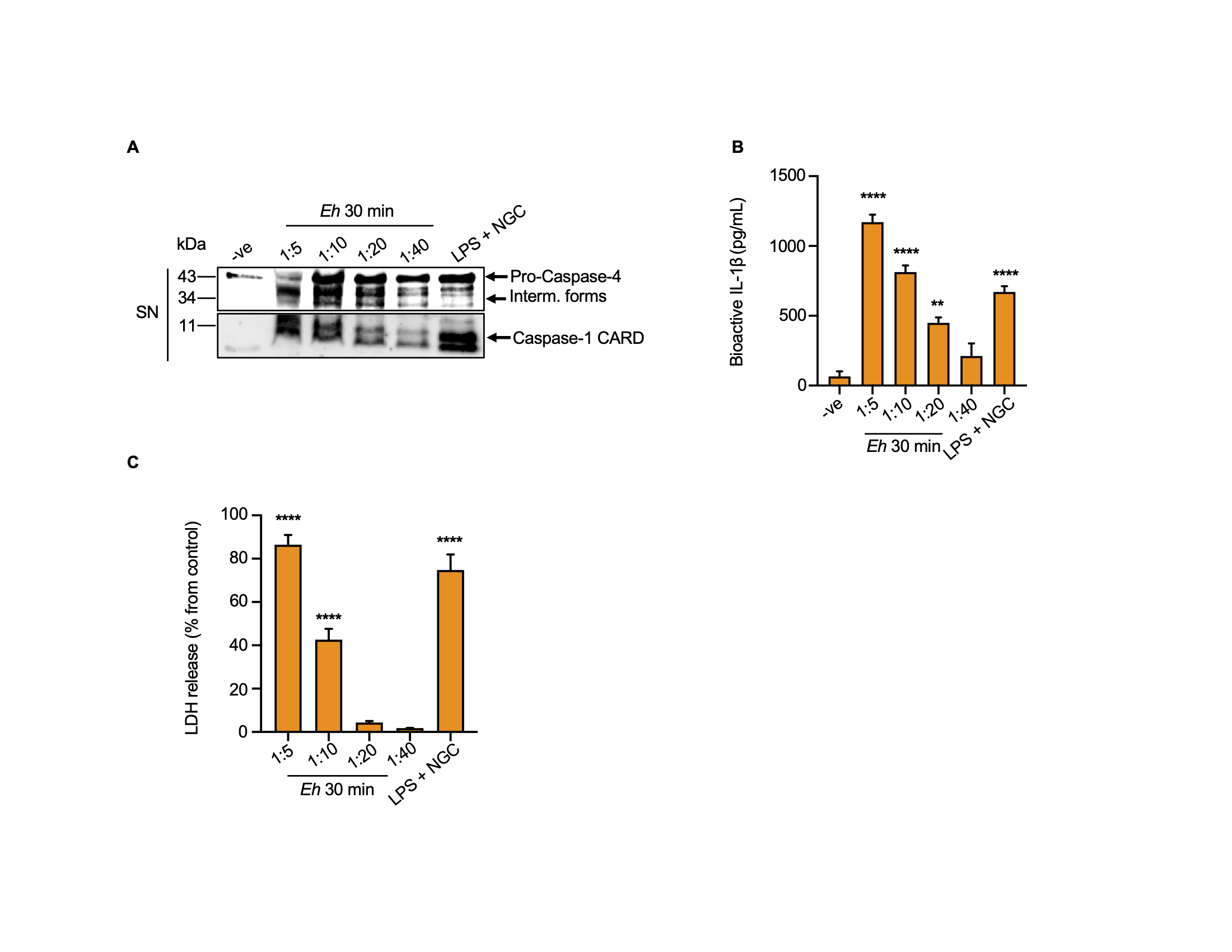

Supplement: S1 Fig — (A) Macrophages were incubated with increasing Eh-macrophage ratios using LPS + NGC as a positive control. Unstimulated macrophages were used as a negative control. Cell supernatant (SN) was TCA precipitated and equal amount of proteins was resolved on SDS-PAGE following the investigation of activated caspase-4 and caspase-1 that was secreted into cell supernatant. (B) Cell supernatant from macrophages was added to HEK-Blue reporter cells to detect bioactive IL-1β via the SEAP assay macrophages that were incubated with Eh for increasing Eh-macrophage ratios. (C) Cell death was also determined by LDH released into cell culture and is shown as a percentage of LDH release compared to non-stimulated cells. Data and immunoblots are representative of at least three independent experiments (n = 3) and statistical significance was calculated with an ANOVA and Bonferroni’s post-hoc test between each Eh-macrophage ratio and positive control treatment, (**p < 0.01, ****p < 0.0001). Bars represent mean ± SEM. (TIFF) [file ppat.1010415.s001.tiff]

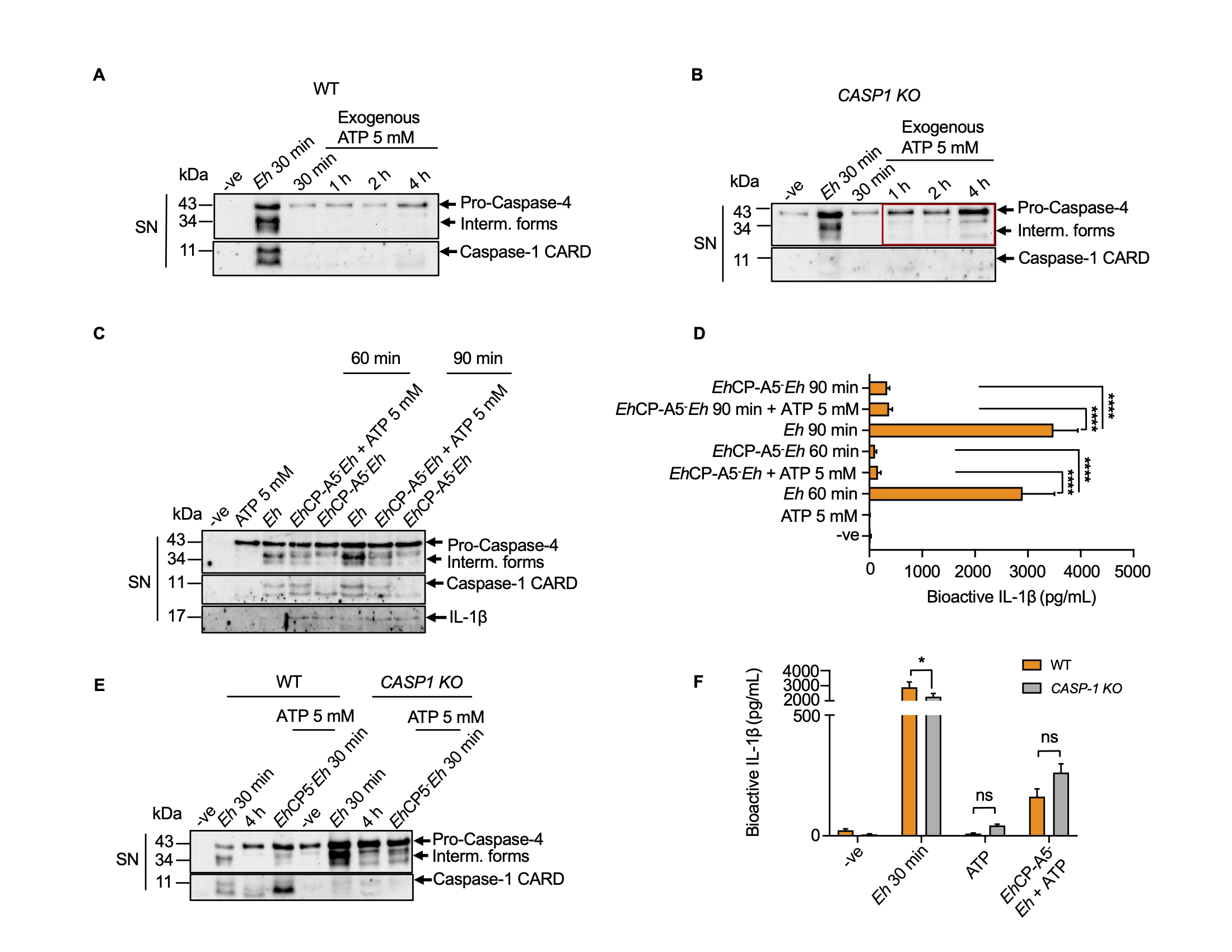

Supplement: S2 Fig — (A, B) Both WT and CRISPR/Cas9 CASP-1 KO macrophages were incubated with 5 mM exogenous ATP from 30 min to 4 h. Restored activation of caspase-4 was detected in CASP1 KO cells stimulated with ATP for 4 h (red box). (C, D) Immunoblot analysis was performed for active caspase-4 and caspase-1 products and IL-1β assay in HEK-Blue reporter cells from macrophages stimulated for 60 or 90 min with WT Eh and EhCP-A5−Eh, respectively. Statistical significance was calculated between each treatment at the same time points (E, F) Exogenous ATP slightly restored caspase-4 activation to rescue IL-1β secretion in the absence of caspase-1 in response to EhCP-A5-Eh. Cell supernatant was TCA precipitated and equal amount of supernatants (SN) was loaded onto SDS-PAGE and immunoblot analysis was performed for caspase-4, caspase-1 and IL-1β. IL-1β assay in HEK-Blue reporter cells from both WT and CASP1KO macrophages was quantified and statistical significance was calculated between each treatment under the same cell types. Data and immunoblots are representative of at least three independent experiments (n = 3) and statistical significance was calculated with one-way ANOVA, followed by Bonferroni’s post-hoc test, (*p < 0.05, ****p < 0.0001, ns: not significant). Bars represent mean ± SEM. (TIFF) [file ppat.1010415.s002.tiff]

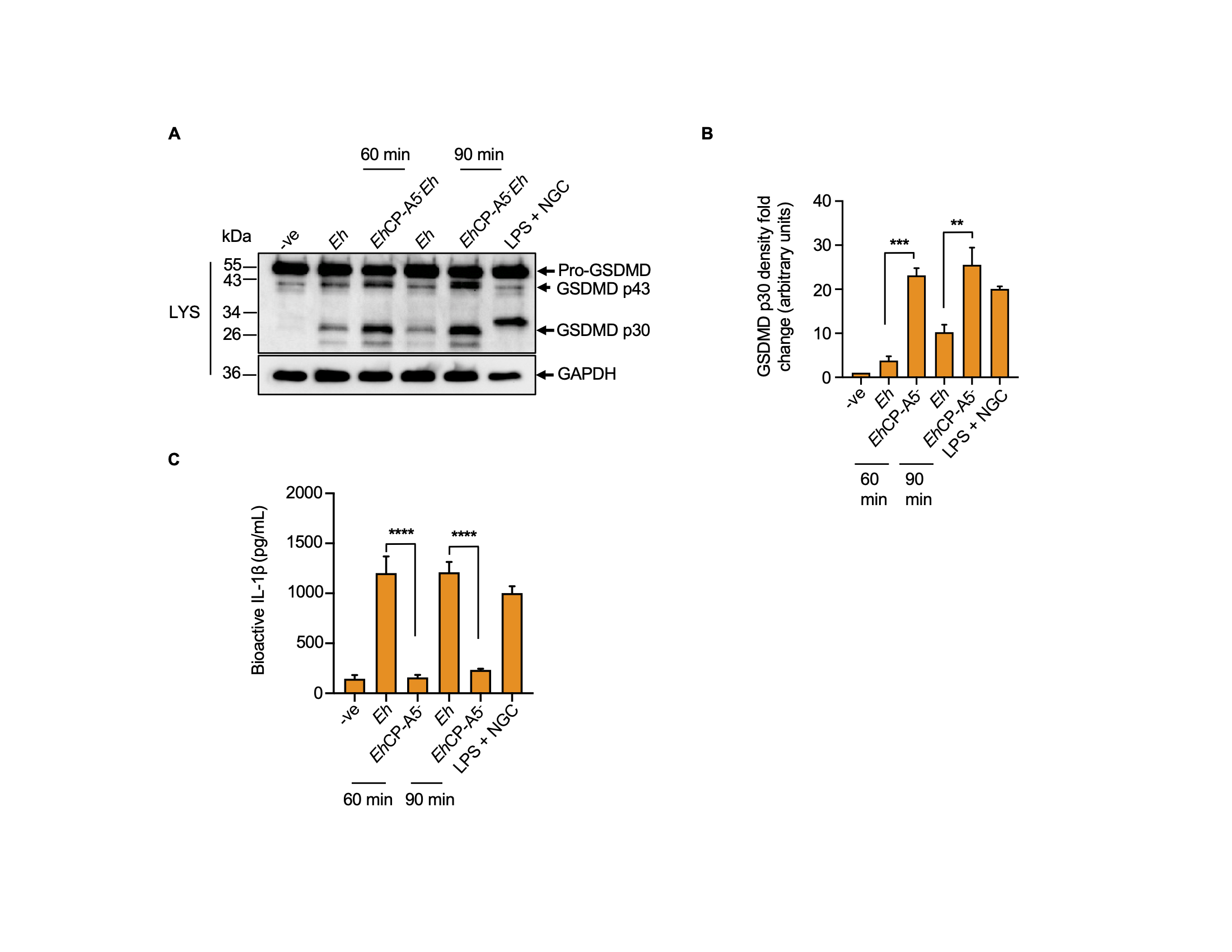

Supplement: S3 Fig — (A) WT THP-1 macrophages were incubated with EhCP-A5-Eh and WT Eh for 60 and 90 min, respectively. Cells were washed and lysed and equal amount of cell lysates was loaded onto SDS-PAGE and immunoblot analysis was conducted to investigate GSDMD cleavage. (B) GSDMD p30 fragment presented in the cell lysate was confirmed with densitometry quantification. (C) IL-1β secretion was quantified in HEK-Blue reporter cells. Data and immunoblots are representative of at least three independent experiments (n = 3) and statistical significance was calculated with one-way ANOVA and post hoc Bonferroni test between WT Eh and EhCP-A5−Eh, (**p < 0.01, ***p < 0.001, ****p < 0.0001). Bars represent mean ± SEM. (TIFF) [file ppat.1010415.s003.tiff]

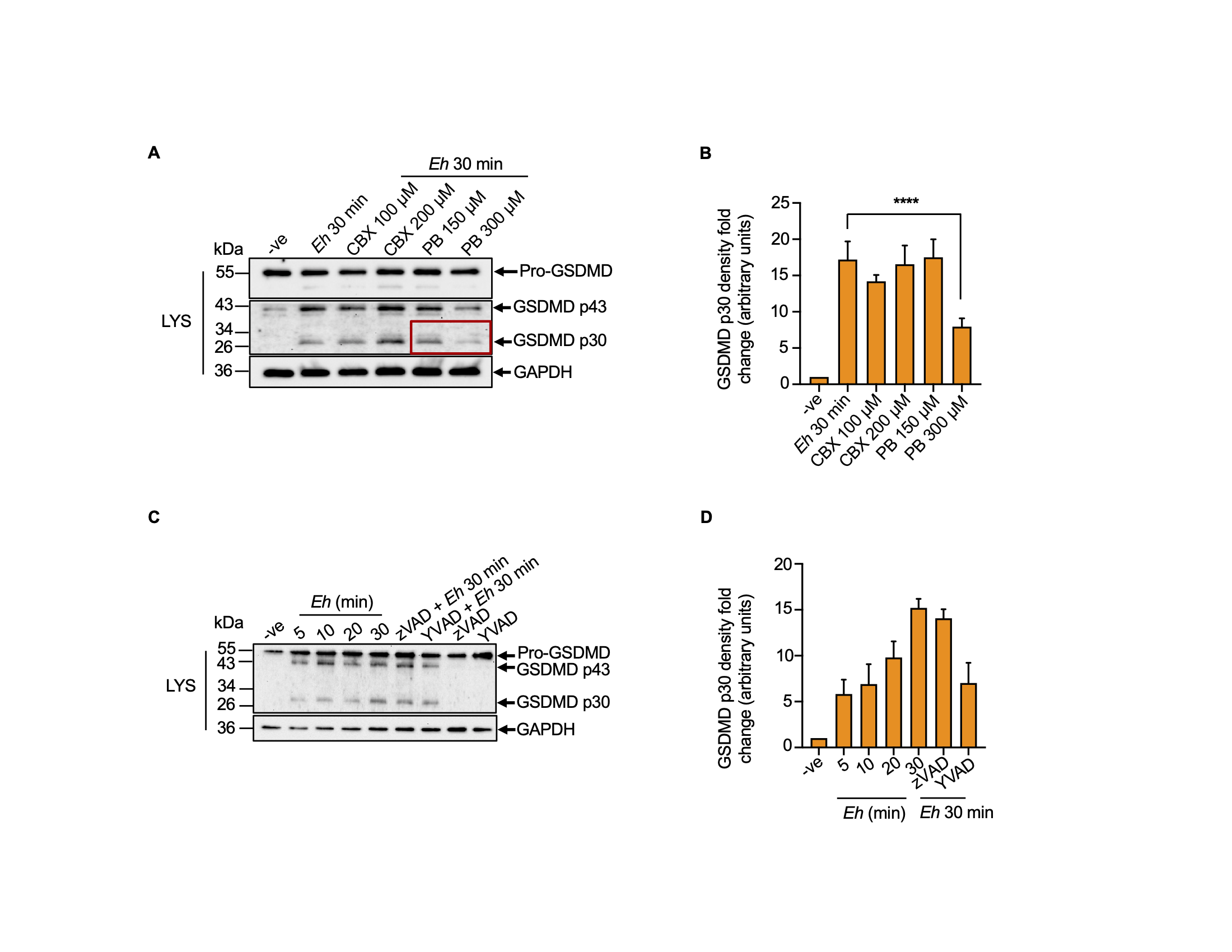

Supplement: S4 Fig — (A, B) Macrophages were pretreated with carbenoxolone (CBX), a connexin/pannexin channel dual inhibitor, or with the pannexin channel inhibitor probenecid (PB) for 30 min prior to Eh stimulation. Cells were washed and lysed and equal amount of cell lysates was loaded onto SDS-PAGE and immunoblot analysis was performed for GSDMD p30 cleaved fragment. (C, D) Macrophages were pre-incubated with the pan-caspase inhibitor Z-VAD-fmk (100 μM) and caspase-1 specific inhibitor Z-YVAD-fmk (100 μM) for 45 min followed by stimulation with Eh (20:1 ratio) for 30 min. The caspase inhibitors were also used alone to tests whether it had an effect on GSDMD cleavage. GSDMD cleaved fragments in the cell lysate were assessed via immunoblotting. Western blots and densitometric analysis are representatives of at least three independent experiments (n = 3). Statistical significance was calculated with one-way ANOVA and post hoc Bonferroni test between Eh stimulation for 30 min and with the addition of inhibitors, (****p < 0.0001). Bars represent mean ± SEM. (TIFF) [file ppat.1010415.s004.tiff]

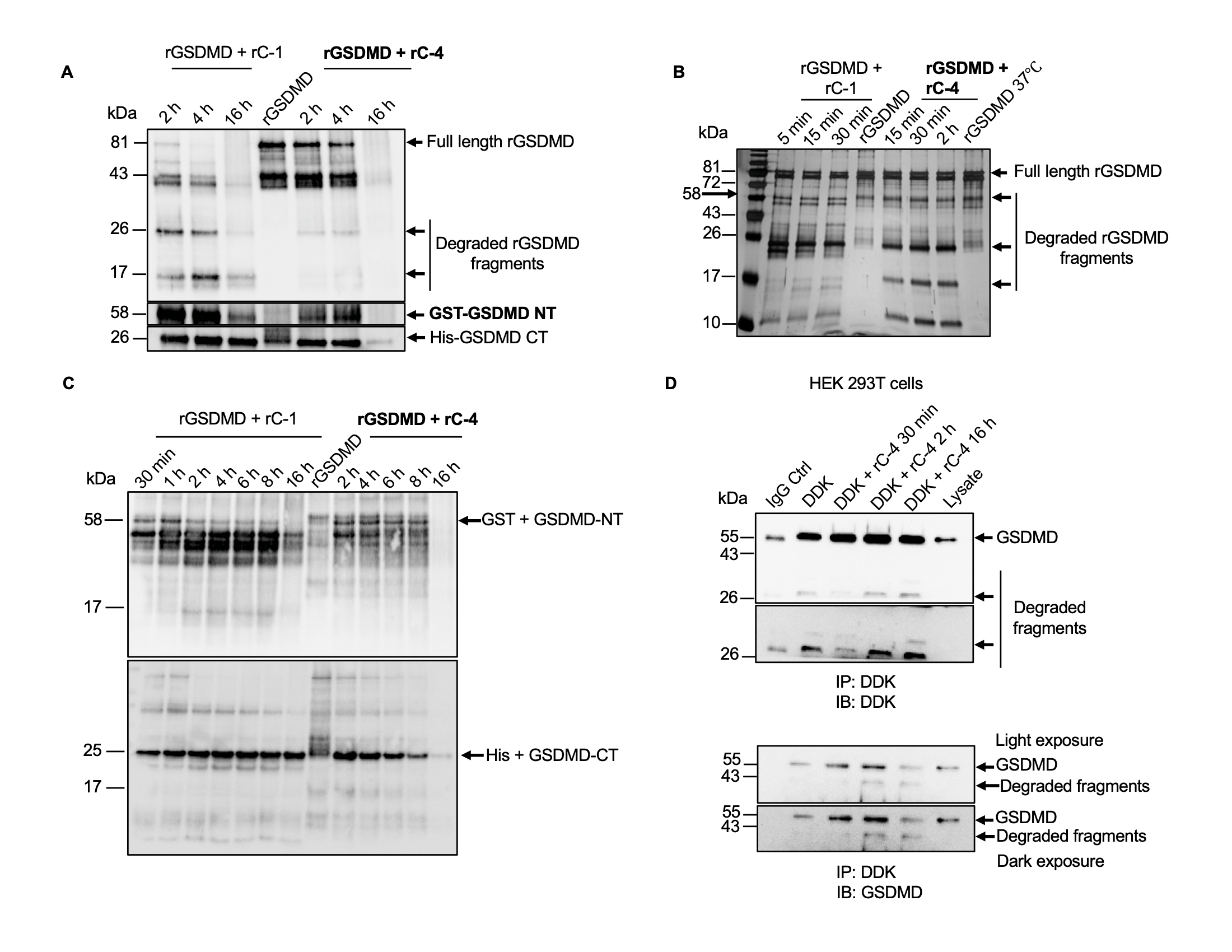

Supplement: S5 Fig — (A) rGSDMD was incubated with active rC-1 and rC-4 for the same amount of time (2 h, 4 h and 16 h) and immunoblot analysis was conducted to assess the degraded fragments of GSDMD. (B) Cleavage of rGSDMD by active rC-1 and rC-4 was conducted at 37°C for indicated incubation time, following by silver staining. rGSDMD was incubated at 37°C for 2 h in the absence of recombinant caspases to investigate if any autoproteolysis exists. (C) Full blots of the cleavage assay conducted on rGSDMD with rC-1 and rC-4 and detected by anti-GST and anti-His antibodies. (D) C terminal Myc-DDk-tagged human GSDMD plasmid was overexpressed in HEK 293T cells and immunoprecipitated with anti-DYKDDDDK antibody. Immunoprecipitants were incubated at 37°C with active rC-4 for various time points (30 min, 2 h and 16 h) and GSDMD cleavage was assessed by western blot with anti-DYKDDDDK and anti-GSDMD antibody. Direct cell lysate was used as a control. Immunoblots are representative of at least three separate experiments (n = 3). (TIFF) [file ppat.1010415.s005.tiff]

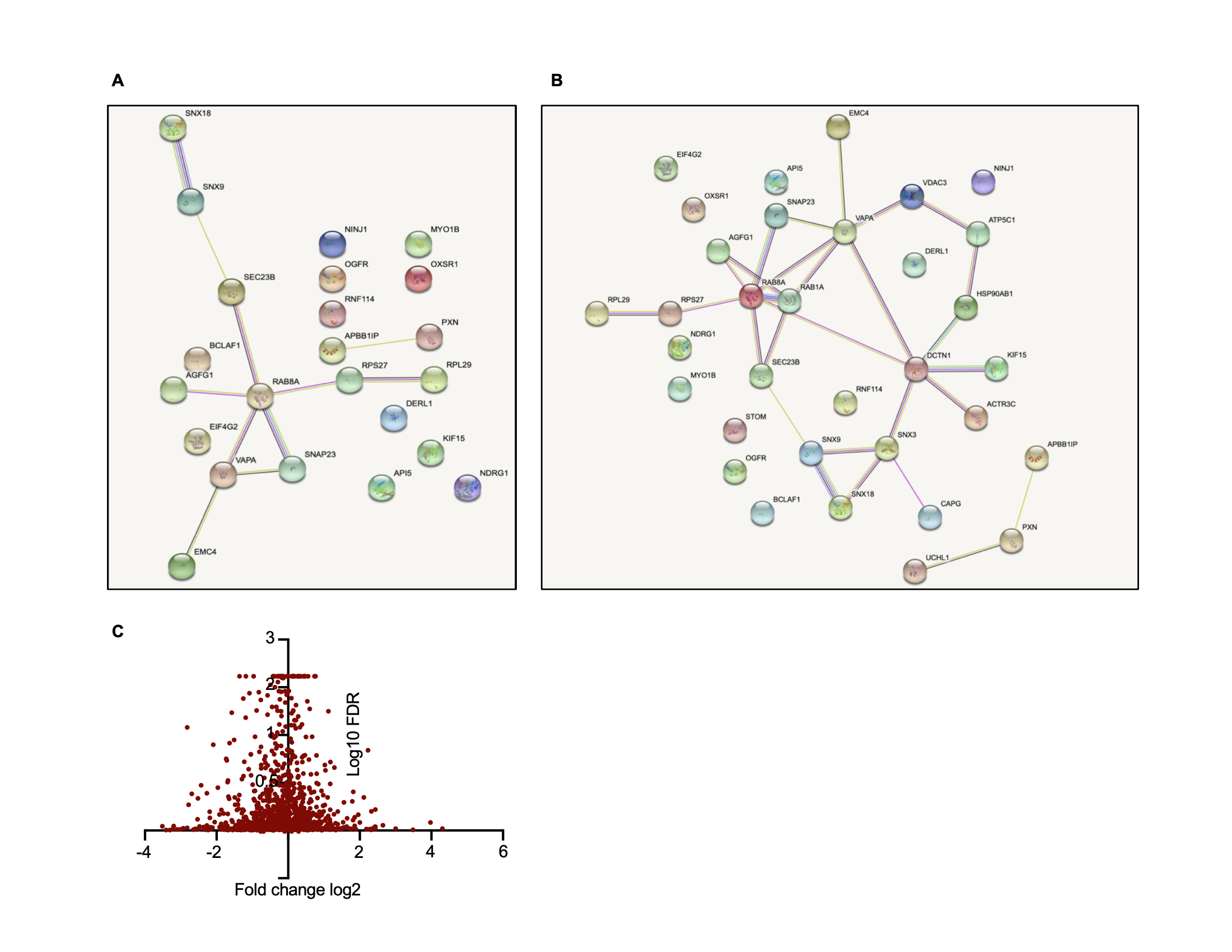

Supplement: S6 Fig — Based on the proteomics analysis in Eh-induced hyperactivated macrophages, a protein-protein interaction assay was conducted by STRING protein-protein interaction analysis. (A) Shown is the network of enriched terms within the downregulated pathway (Apoptotic signaling pathway and membrane trafficking), where connections that share the same cluster typically interact to each other. (B) Network demonstrating the interactions between downregulated pathway and upregulated pathway (Regulation of proteolysis and secretion by cell) in hyperactivated macrophages by STRING analysis. (C) Volcano plot of proteins from both downregulated and upregulated pathways in Eh-induced hyperactivated macrophages with log2 interpretation. (TIFF) [file ppat.1010415.s006.tiff]
